# The dPix-Git complex is essential to coordinate epithelial morphogenesis and regulate myosin during *Drosophila* egg chamber development

**DOI:** 10.1371/journal.pgen.1008083

**Published:** 2019-05-22

**Authors:** Lucas G. Dent, Samuel A. Manning, Benjamin Kroeger, Audrey M. Williams, Abdul Jabbar Saiful Hilmi, Luke Crea, Shu Kondo, Sally Horne-Badovinac, Kieran F. Harvey

**Affiliations:** 1 Peter MacCallum Cancer Centre, Melbourne, Victoria, Australia; 2 Sir Peter MacCallum Department of Oncology, University of Melbourne, Parkville, Victoria, Australia; 3 Department of Anatomy and Developmental Biology, and Biomedicine Discovery Institute, Monash University, Clayton, Australia; 4 Department of Molecular Genetics and Cell Biology, The University of Chicago, Chicago, IL, United States of America; 5 Laboratory of Invertebrate Genetics, National Institute of Genetics, Yata, Mishima, Shizuoka, Japan; NYU School of Medicine, UNITED STATES

## Abstract

How biochemical and mechanical information are integrated during tissue development is a central question in morphogenesis. In many biological systems, the PIX-GIT complex localises to focal adhesions and integrates both physical and chemical information. We used *Drosophila melanogaster* egg chamber formation to study the function of PIX and GIT orthologues (dPix and Git, respectively), and discovered a central role for this complex in controlling myosin activity and epithelial monolayering. We found that Git’s focal adhesion targeting domain mediates basal localisation of this complex to filament structures and the leading edge of migrating cells. In the absence of *dpix* and *git*, tissue disruption is driven by contractile forces, as reduction of myosin activators restores egg production and morphology. Further, *dpix* and *git* mutant eggs closely phenocopy defects previously reported in *pak* mutant epithelia. Together, these results indicate that the dPix-Git complex controls egg chamber morphogenesis by controlling myosin contractility and Pak kinase downstream of focal adhesions.

## Author summary

A major challenge in biology is to identify the genes and processes that build tissues of correct shape and function. Recently, transmission of mechanical forces through cell adhesions, and control of cell tension via contractile force-generating proteins, have emerged as fundamental to tissue development. Currently, we do not understand how these separate processes are integrated. We gained new insight into morphogenesis and control of contraction through adhesion-localised proteins, by studying mutants of the dPix-Git focal adhesion complex in *Drosophila melanogaster* egg chambers, a 3D model of tissue morphogenesis. We found that the dPix-Git complex is essential to maintain cell monolayers, and is a regulator of the contractile force-generating protein, myosin. In the absence of the dPix-Git complex, irregular myosin activation led to tissue disruption, however modest suppression of myosin activators rescued this defect. Remarkably, the dPix-Git complex is essential for egg chamber development, but appears dispensable for other *D*. *melanogaster* epithelia, indicating the mechanisms that couple adhesion signalling and cell contractile forces are tissue specific. Our study reveals a key molecular link between cell adhesions and contraction, and the conservation of the dPix-Git module in metazoans suggests this mechanism is likely to be evolutionarily conserved in other tube-like tissues.

## Introduction

**Fig 1:**
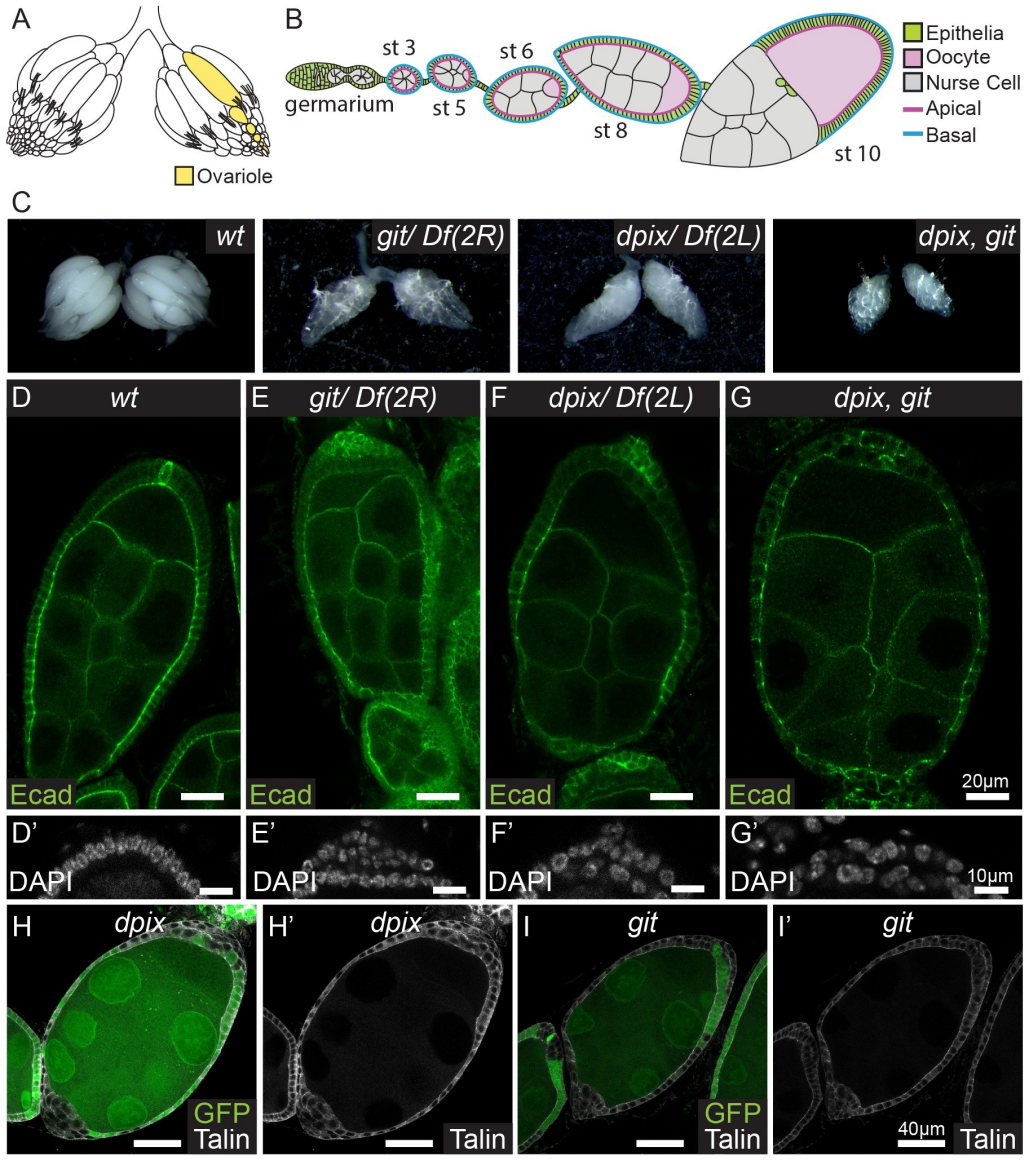
The dPix-Git complex is required cell autonomously for epithelial morphogenesis during *Drosophila melanogaster* egg chamber development. **(A-B)** Schematic diagrams of an adult *D*. *melanogaster* ovary with an ovariole structure highlighted (yellow) (A), and an individual ovariole containing egg chambers up to stage 10 (B). Key tissue types are follicular epithelia (green), oocyte (pink), and germline nurse cells (grey). **(C)** General appearance of adult *D*. *melanogaster* ovaries of the indicated genotypes: from left to right, wild-type (*wt*); *git*; *dpix*; *dpix*, *git*. **(D-G’)** Stage 7–8 egg chambers stained with E-Cadherin (Ecad), and enlarged projections of the posterior tip of egg chambers stained with DAPI (D’, E’, F’, G’) for the following genotypes: (D-D’) wild-type; (E-E’) *git*; (F-F’) *dpix*; (G-G’) *dpix*, *git*. Scale bars 20 μm for (D, E, F, G) and 10 μm for (D’, E’, F’, G’). **(H-I’)** *D*. *melanogaster* egg chambers with a mosaic of wild*-*type and *dpix* (H-H’) or *git* (I-I’) mutant tissue. In each egg chamber wild-type tissue is labelled by GFP, whereas *dpix* and *git* mutant tissue lacks GFP. Cells are visualised with Talin antibody (grey). Scale bars 40 μm.

Organogenesis requires the coordinated integration of biochemical and mechanical information at the level of cells and their neighbours, as well as across entire tissues [1,2]. *Drosophila melanogaster* (*D*. *melanogaster*) egg chamber development has emerged as a powerful system for identifying myriad developmental processes, and understanding how they interact at the cell and tissue scales [3–5]. Egg chambers originate from stem cells in a structure called the germarium (Fig 1A and 1B), and progress through 14 stages of development. New egg chambers continually ‘bud’ from the germarium and remain connected by stalk structures, like ‘beads on a string’ (Fig 1A and 1B). These egg chambers consist of a monolayer of somatic follicular epithelial cells encapsulating a cyst of growing germline cells. Egg chamber follicular epithelial cells secrete basement membrane proteins to form an extracellular matrix (ECM), which surrounds the growing organ. Over a period of days the egg chamber grows by approximately three orders of magnitude. This growth is preferentially channeled along the egg chamber’s anterior-posterior axis to form a 2–3 fold elongated ellipsoid of approximately 850 cells, which establishes the foundations of the embryonic body plan. During this period of growth and elongation the somatic follicle cells maintain apical-basal polarity [6] and tissue monolayering to preserve egg chamber function and integrity [7–9].

A series of studies have revealed strict spatial and temporal regulation of the diverse roles of non-muscle myosin II (myosin), a contractile force-generating protein, during egg chamber development [10–15]. Amongst other roles, apical myosin provides mechanical resistance to pressure generated by the growing germline cyst early in egg development [14], and drives cell contraction and tissue elongation at the early stages of egg chamber maturation [15]. Later in development, basal myosin oscillations are deployed to directionally constrict growth along the anterior-posterior axis [10]. Landmark studies have also revealed that during early stages (stages 1–8), follicle cells undertake collective epithelial sheet migration and this migration is required for egg chamber elongation [16,17]. Collective cell migration relies on the formation of actin-based protrusions at the leading edge of each follicle cell and contributes to elongation by generating tissue level polarity in basal actin and extracellular matrix fibres [16–18].

Previous genetic studies of egg chamber development identified a similar set of defects in cells lacking different focal adhesion proteins (which engage the ECM), and the sterile-20 kinase p21-activated kinase (Pak). These defects include loss of monolayering [7,19], irregularities in the ordering of basal actin filaments [20–22], and altered myosin activity [10,22]. In addition, in a range of cell culture and animal systems, signal transduction from focal adhesions has been linked to the activity of p21-activated kinase family (PAK) proteins via the oligomeric PIX-GIT complex which includes the P21-activated kinase interacting exchange factor (PIX) proteins, and the G-protein coupled receptor kinase interacting proteins (GIT) [23–25]. PIX proteins are RhoGEFs, and GIT proteins are ArfGAPs, and each homodimerise and heterodimerise to form the PIX-GIT signalling scaffold [26–29]. The PIX-GIT complex binds numerous proteins at a range of subcellular locations. Prominent amongst these, PIX and GIT have been identified as part of the ‘core’ integrin based adhesome [30]. In this context, GIT targets the entire complex to focal adhesions [31], while PIX in turn recruits PAK to focal adhesions for regulation of activation [32,33].

In mammals, the *PIX* and *GIT* genes are each duplicated, giving rise to *α-PIX*/*β-PIX*, and *GIT1*/*GIT2*, generating the potential for redundancy and a practical barrier to genetic studies. In contrast, *D*. *melanogaster* has a single copy of each gene, making it an ideal system to interrogate their function. In *D*. *melanogaster* the *PIX* and *GIT* orthologues are referred to as *dpix* and *git* respectively. *D*. *melanogaster* studies have revealed that dPix and Git have roles that are distinct from each other in the context of neuronal function [34,35], whilst they appear to co-operate during muscle morphogenesis [36] and also work together to control Hippo pathway dependent tissue growth [37]. To further understand the role of the dPix-Git signalling module in organogenesis, we examined *dpix* and *git* mutant *D*. *melanogaster* and focused on defects that were common to both mutations. *dpix* and *git* were each required during egg chamber development for cell intercalation, correct myosin activation, and to maintain a follicular epithelial monolayer. In the absence of *dpix* and *git*, aberrant myosin activation generated force anisotropies that led to cell deformation and loss of epithelial integrity. Interventions designed to mildly suppress myosin activity were sufficient to rescue egg production and elongation defects. The dPix-Git complex was predominantly basally localised, with a planar polarised enrichment at basal filament structures toward the leading edge of cells undergoing collective migration. Altogether, this study identifies a tissue specific and essential requirement for the dPix-Git complex in egg chamber development.

## Results

### dPix and Git are each required for egg development

To further understand the role of the dPix-Git signalling module in organ development, we examined *dpix* (CG10043, also known as *rtgef*) [34] and *git* (CG16728) mutant [36] *D*. *melanogaster* and looked for shared developmental defects. As previously reported, homozygous mutations for *dpix* and *git* were each semi-lethal [34,36]. Surviving adults emerged with ‘crumpled’ wings but were otherwise grossly morphologically normal (S1A Fig). Notably, both *dpix* and *git* adult females produced very few eggs. This prompted examination of *dpix* and *git* ovaries and revealed that while early stage egg chambers were present, these generally did not progress to maturity (Fig 1C). To test if *dpix* and *git* function redundantly in egg chamber development we generated *dpix*, *git* double mutant animals. These animals were also semi-viable and eclosed with crumpled wings (S1A Fig), but produced no mature eggs (Fig 1C). This indicates that the dPix-Git signalling complex is essential for egg development, and the more severe phenotype in double mutants compared to each single mutant suggests that *dpix* and *git* have partially independent roles. Taken together, these results show that dPix and Git are essential regulators of egg production, whereas they appear to be dispensable for the development of most *D*. *melanogaster* organs.

### dPix and Git regulate epithelial monolayering cell autonomously

To understand the developmental processes that are misregulated in the absence of *dpix* and *git*, we studied the anatomy of mutant egg chambers and found that monolayering of the follicular epithelium was disrupted from as early as stage 3 (Fig 1D–1G’ and see S1B–S1B” Fig). Markers of cell polarity such as E-cadherin were both elevated and mislocalised in supernumerary follicular epithelial layers (Fig 1D–1G and S1C and S1D Fig). In particular, while E-cadherin was apically enriched and also present on lateral junctions in wild-type tissue, it was distributed around the entire cell in the extra follicular epithelium layers of *dpix* and *git* mutants. This appears similar to the loss of cell polarity seen in extra follicular epithelium layers of *α-spectrin* mutant animals [9].

We used the FLP/FRT system to generate clones of *dpix* or *git* mutant tissue in otherwise heterozygous animals, and found that loss of *dpix* or *git* causes multilayering of the follicular epithelium (Fig 1H–1I’), even in small clones (S1G Fig), indicating that the requirement for *dpix* and *git* is autonomous to the follicle cells. Interestingly, while PIX and GIT proteins are known to influence focal adhesion turnover and maturation in cell culture systems [38–41], we did not see an obvious effect on the accumulation of the focal adhesion component talin in monolayered *dpix* or *git* mutant tissue (Fig 1H–1I’), however talin levels were often increased in multilayered tissue (S1E and S1F Fig). We did not see a general disruption of apical-basal polarity in monolayered clones, as indicated by unchanged apical localisation of β-heavy-spectrin in *dpix* clones (S1H–S1H’ Fig).

**Fig 2:**
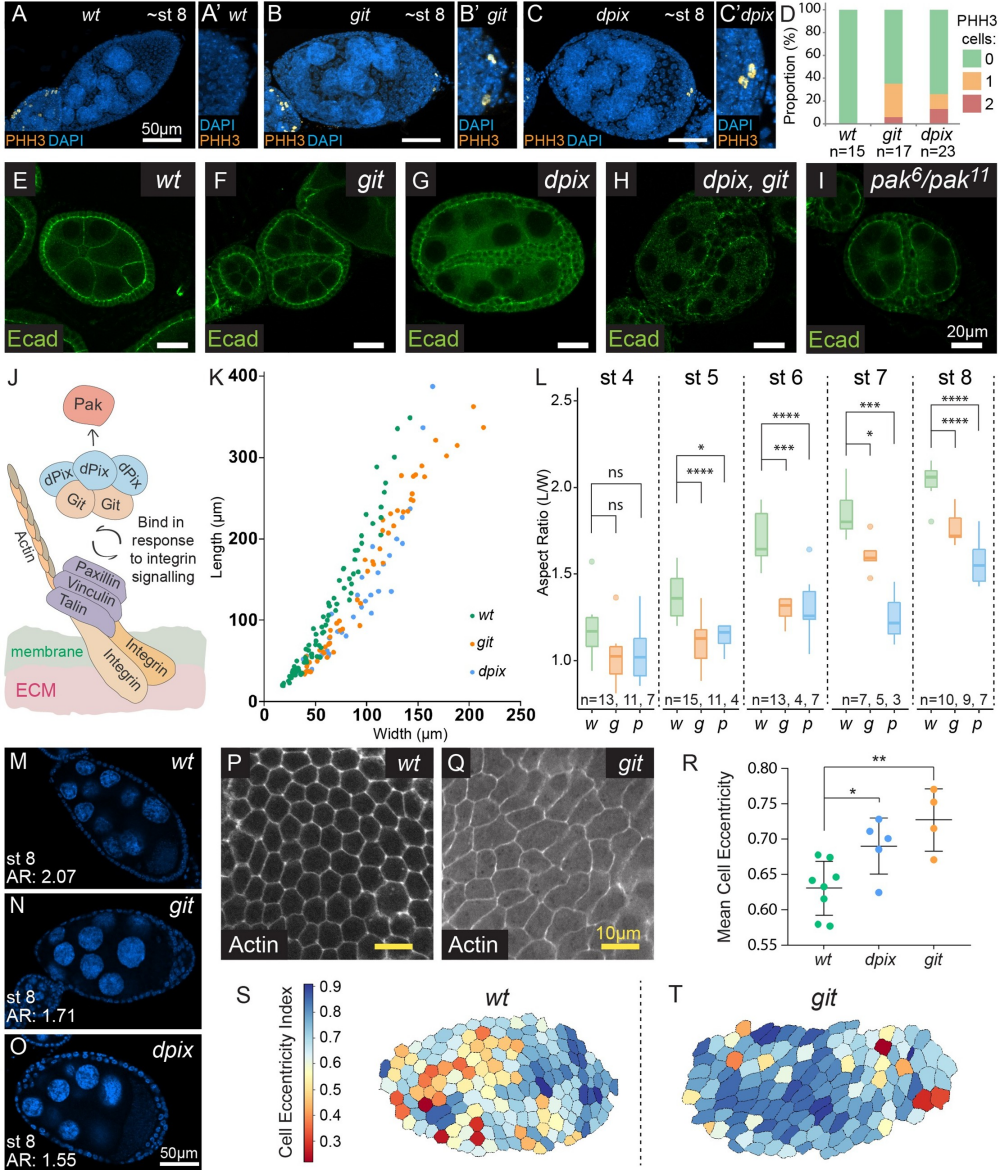
Loss of *dpix* or *git* leads to ectopic cell division in multilayered tissues, phenocopies loss of *pak*, and disrupts egg chamber shape, and cell shape. **(A-C’)** Maximum intensity projections of wild-type (*wt*) (A-A’), *git* (B-B’) and *dpix* (C-C’) stage 8 egg chambers visualised with DAPI (blue) and stained with phospho-Histone H3 (PHH3) (orange). (A, B, C) show whole egg chambers, and (A’, B’, C’) show enlargements of the posterior region. Scale bars are 50 μm. **(D)** Quantification of the frequency of PHH3 positive cells in stage 7–9 egg chambers for the indicated genotypes. **(E-I)** Wild-type egg chamber and mutant egg chambers displaying side-by-side egg chamber fusion defects. Genotypes are: *wt* (E); *git* (F); *dpix* (G); *dpix*, *git* (H); *pak* (I). Scale bars 20 μm. **(J)** Schematic of the dPix-Git complex highlighting the conserved interaction with Pak (via the SH3 domain of dPix), and tension dependent basal localisation at focal adhesions (via Git’s Paxillin binding domain). **(K)** Scatter plot of egg chamber width against length for *wt* (green), *git* (orange) and *dpix* (blue) genotypes between stages 3 and 10. **(L)** Box plot representation and statistical comparison of egg chamber aspect ratio between stages 4–8 of egg chamber development. Genotypes: *w* = wild-type; *g* = *git*; *p* = *dpix*. Statistical tests are ANOVA with post hoc Tukey’s test, and significance is: * = p < 0.05; *** = p < 0.001; **** = p < 0.0001; ns = not significant. **(M-O)** Examples of stage 8 egg chambers from *wt* (M), *git* (N), and *dpix* (O) egg chambers, with aspect ratio (AR) displayed. Visualised with DAPI (blue). Scale bars 50 μm. **(P-Q)** Cortical actin stains at the basal surface of wild-type and *git* mutant stage 7–8 epithelia. Scale bars 10 μm. **(R)** Quantification of mean cell eccentricity in wild-type, *dpix* and *git* mutant stage 7–8 egg chambers. Error bars are standard deviation. Statistical test is ANOVA with post hoc Dunnett’s test. Each ‘n’ represents a separate egg chamber, with a continuous area of at least 40 cells measured per egg chamber. Sample sizes are: *wt*, n = 8; *dpix*, n = 5; *git*, n = 4. Significance: * = p < 0.05; ** = p < 0.01. **(S, T)** Choropleth map of wild-type and *git* epithelia, with each cell coloured according to the degree of eccentricity.

Follicular epithelium cells are mitotic until stage 6, after which they begin Notch signalling mediated endo-replication [42]. Previous studies of egg chambers have shown that multilayering can be caused by excessive proliferation and re-entry into mitosis [9,43–45], therefore we assessed the presence of mitotic cells in *dpix* and *git* homozygous mutants using phospho-Histone H3 staining. We observed no increase in proliferation before stage 6 (S2A Fig), but found prolonged cell divisions occurring in the already multilayered tissues of *dpix* and *git* egg chambers between stages 7 and 9 (Fig 2A–2D and S2A and S2B Fig). This raised the possibility that Notch signalling was defective. To investigate this we stained for Eyes Absent, which is normally suppressed by Notch signalling after stage 6 [46], and found increased expression in ectopic cell layers (S2C–S2G” Fig). FasIII is another protein suppressed by Notch after stage 6 [47], and was also increased in multilayered cells (S2H–S2J” Fig). The restriction of ectopic proliferation and Eyes Absent expression to multilayered cells suggests that Notch deregulation and ectopic proliferation are a consequence of multilayering which may exacerbate this phenotype, but are not likely to cause epithelial multilayering in *dpix* and *git* mutants.

### *dpix* and *git* egg chambers have defects in cell intercalation, cyst separation, and phenocopy *pak* mutants

In addition to multilayering of the follicular epithelium we observed multiple cell intercalation defects in *dpix* and *git* mutants. First, both *dpix* and *git* mutants showed intercalation defects in the stalk structures that joined consecutive egg chambers (S3A–S3C Fig). Second, we found compound egg chambers containing more than 15 germline cells (S3D–S3F Fig), a defect that has previously been associated with disrupted migration of pre-follicular cells between germline cysts [48]. Finally, we observed egg chamber fusions in some ovarioles (Fig 2E–2I) which we confirmed as side-by-side fusion of egg chambers by oocyte staining (S3G–S3H” Fig). The side-by-side fusion phenotype is very reminiscent of a myosin-mediated cell intercalation defect that was previously reported in developing ovaries of *pak* mutant animals [49]. This specific phenocopy of *pak* mutants, combined with the well characterised physical interaction between PIX-GIT and PAK proteins, suggest that a dPix-Git-Pak signalling axis regulates egg chamber morphogenesis (Fig 2J).

### *dpix* and *git* maintain egg chamber shape from early stages of development

In order to understand the effect of *dpix* or *git* mutation on overall egg chamber morphology, we measured the length and width of egg chambers between stages 3 and 10 (Fig 2K). *dpix* and *git* egg chambers with side-by-side fusions or compound germline cysts were excluded from analysis. This allowed us to view the relationship between egg chamber width and length independent of stage, and revealed that *dpix* and *git* mutant egg chambers tended to be wider than wild-type egg chambers of similar length (Fig 2K). Next, we developmentally staged wild-type, *dpix* and *git* egg chambers between stages 4–8 using DAPI based features [50], and compared their aspect ratio (length/width) (Fig 2L). Even from early stages, *dpix* or *git* mutant egg chambers had a reduced aspect ratio compared to wild-type (Fig 2L) and this persisted through to later stages (Fig 2L–2O). The observed effect on aspect ratio from early stages of elongation is consistent with *pak* mutants [15], but contrasts with other mutants that affect egg aspect ratio, such as *lar*, which appear to diverge from wild-type at later stages [20,51].

To investigate whether overall changes in egg chamber shape are associated with cell shape changes, we imaged cortical actin at the basal regions of the follicular epithelium at stage 7–8 (Fig 2P–2Q) and measured a range of cell morphological characteristics (see materials and methods). By stage 7–8, cells in *dpix* and *git* homozygous mutant tissues had greater directional elongation than wild-type (greater eccentricity) (Fig 2R–2T), indicating that loss of *dpix* or *git* at the whole tissue scale produced cell shape distortions within the follicular epithelium.

### dPix and Git maintain epithelial integrity via the regulation of non-muscle myosin II

Non-muscle myosin II (myosin) is a contractile force-generating protein. The C-terminus of myosin molecules can self-associate to form bipolar filaments that simultaneously bind two anti-parallel actin filaments and ‘pull’ them towards one another. When actin filaments are attached to cell membranes via adherens junctions, or to basal membranes via focal adhesions, contraction from myosin generates forces which drive cell movement and shape changes [52]. Two reasons led us to examine the role of dPix and Git in regulating myosin activity. First, the data above suggest that a Git-dPix-Pak signalling axis functions in follicular epithelia, and Pak is known to antagonise myosin activation in this tissue [22,49]. Second, we had seen changes to the elongation and shape of *dpix* and *git* mutant epithelial cells near their basal surfaces (Fig 2P–2T). We considered that these deformations could be caused by deregulated myosin activity, resulting in disorganised cell membrane contractility and tension.

**Fig 3:**
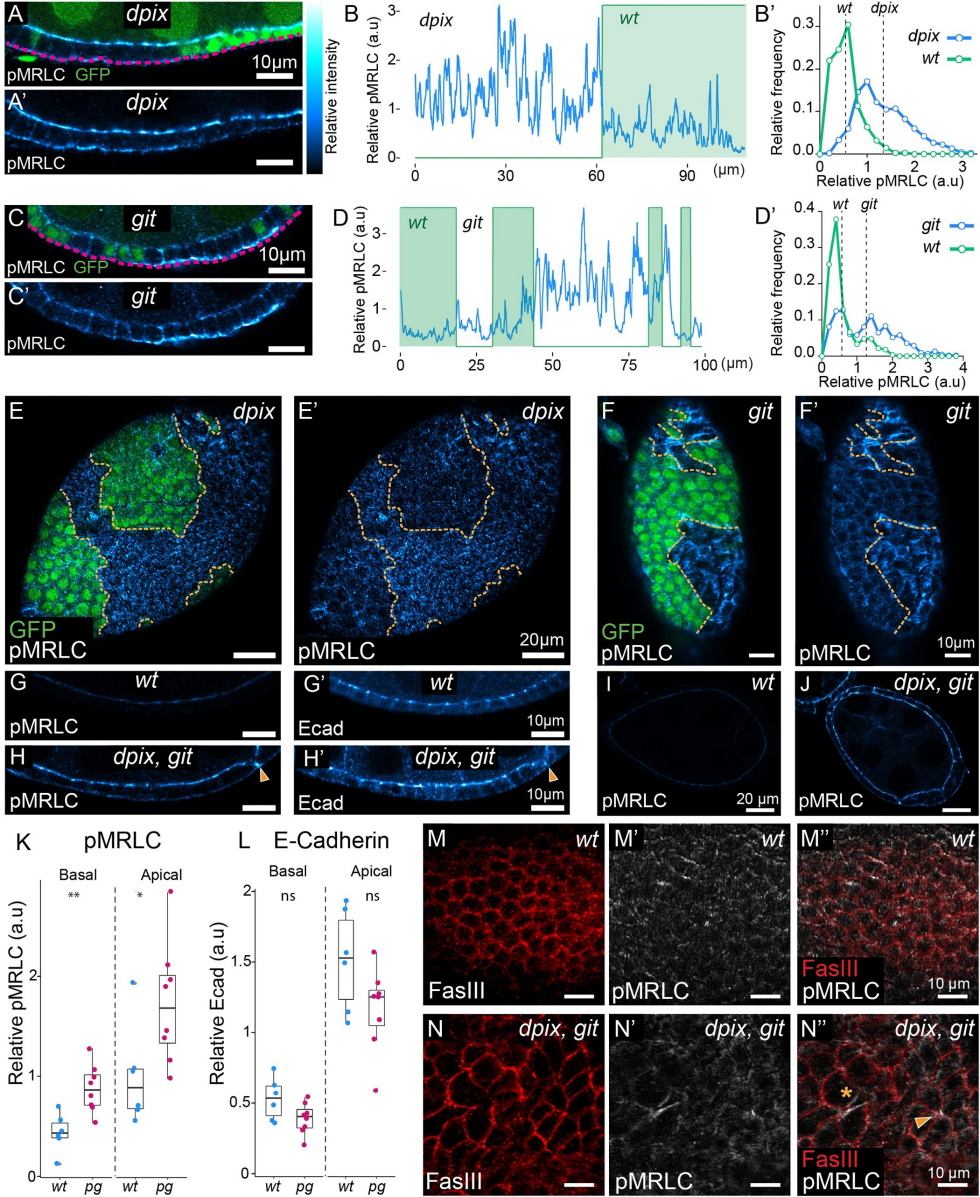
The dPix-Git complex spatially restricts subcellular activation of myosin to maintain tissue integrity during epithelial development. **(A-F’)** Comparison of phosphorylated myosin regulatory light chain (pMRLC), between wild-type and adjacent mutant tissue. For (A, C, E, F) mutant tissue lacks GFP. (A-A’) Cross-section of a stage 6–7 egg chamber, with a mosaic of wild-type and *dpix* tissue. (B-B’) Quantification of pMRLC intensity (B) along a basal transect (magenta line in A) of *dpix* mosaic tissue from (A-A’), and corresponding normalised frequency polygons depicting distribution of pMRLC values in each genotype (B’). Dashed lines in (B’) are average intensity values for the indicated genotype. Bin width = 0.2. (C-C’) Basal cross-section of a stage 6 egg chamber, with a mosaic of wild-type and *git* tissue. (D-D’) Quantification of pMRLC intensity (D) along a basal transect (magenta line in C) of *git* mosaic tissue from (C-C’), and corresponding normalised frequency polygons depicting distribution of pMRLC values in each genotype (D’). Dashed lines in (D’) are average intensity values for the indicated genotype. Bin width = 0.2. (E-E’) Basal pMRLC distribution in a *dpix* mosaic tissue that has maintained monolayering in main body follicle cells. (F-F’) Basal pMRLC distribution in *git* mosaic tissue with multilayering in *git* mutant tissue. Orange lines in (E-F’) indicate genotype boundaries. **(G-J)** Follicular epithelia of stage 6–7 wild-type and *dpix*, *git* double mutant egg chambers co-stained with pMRLC and E-Cadherin (Ecad). (G-G’) wild-type egg chamber. (H-H’) *dpix*, *git* mutant egg chamber. Arrowhead indicates collapsed lateral membrane at the point of pMRLC accumulation in *dpix*, *git* mutant. (I) Tissue scale cross section of pMRLC signal from wild-type egg chamber in (G-G’). (J) Tissue scale cross section of pMRLC signal from *dpix*, *git* egg chamber in (H-H’). Scale bars 10 μm (G-H’), and 20 μm (I-J). **(K-L)** Quantification of apical and basal pMRLC, and E-Cadherin in wild-type and *dpix*, *git* double mutant follicular epithelia. Genotypes and sample sizes are: wild-type (*wt*), n = 6 egg chambers; *dpix*, *git* (*pg*), n = 8 egg chambers. Statistical analyses are Welch’s unequal variances t-tests. Significance: * = p < 0.05; ** = p < 0.01; ns = not significant. **(M-N”)** Basal sections of ~stage 7 wild-type (M-M”) and *dpix*, *git* (N-N”) egg chambers. Membranes are marked by fasciclin III (FasIII) and activated myosin is marked by pMRLC. Purse-string like accumulation of pMRLC is indicated by asterisk, and aberrant-cell junction indicated by arrowhead (N”). Relative intensity index applies to pMRLC and Ecad signal in (A-A’, C-C’, E-J).

To test for a cell autonomous defect in myosin activation we stained *dpix* or *git* mosaic egg chambers with antibodies against phosphorylated Ser19 (Ser21 in *D*. *melanogaster*) of myosin regulatory light chain (pMRLC), and examined the intensity and distribution of this activated form of myosin. We examined *dpix* or *git* clones that had maintained monolayering and found increased myosin activation at the basal membrane compared to neighbouring wild-type cells (Fig 3A–3E’). While myosin was hyperactivated in both *dpix* and *git* clones, we saw some evidence of non-cell autonomous effects at clone boundaries which may be caused by the ability of altered mechanical properties in one cell to affect myosin in neighbouring cells [53]. Additionally, we also observed a cell autonomous increase in pMRLC at the basal membranes of cells in clones which had resulted in epithelial multilayering (shown for *git* in Fig 3F–3F’).

To understand how removal of the entire dPix-Git complex affects myosin activation we examined *dpix*, *git* double mutants and found that pMRLC was increased at both the apical and basal membranes of follicular epithelial cells (Fig 3G–3J). To avoid complications from loss of apicobasal cell polarity, as has been reported in *pak* mutants [54], we only examined regions of mutant epithelia that had maintained monolayering. We quantified these observations and found an approximately 2 fold increase in pMRLC at basal membranes, and a 1.5–2 fold increase in pMRLC signal at the apical membrane (Fig 3K). To determine if the effect on myosin was specific, and as a control for optical sectioning of the tissue, we also imaged E-cadherin and found no significant difference in apical or basal cell regions (Fig 3L). Therefore, loss of both *dpix* and *git* led to myosin activation and this could not be explained by a general effect of enrichment of proteins at membranes or cell junctions. The basal deregulation of pMRLC in *dpix* or *git* clones, compared with a combined basal and apical deregulation of pMRLC in *dpix*, *git* homozygous mutants suggests that the basal deregulation of pMRLC is a primary cell biological defect of disrupting the dPix-Git complex, while the apical misregulation of pMRLC may be a secondary effect of alterations in tissue wide morphogenetic processes.

We also found cells in *dpix*, *git* double mutants where activated myosin was localised to lateral cell junctions and where this loading of activated myosin was associated with almost complete contraction of the length of lateral junctions (Fig 3H–3H’ and 3J, and arrow in 3H–3H’). Imaging optical sections across the basal surface of *dpix*, *git* double mutants suggested that the de-regulation of myosin contractility generated force anisotropies that resulted in disorganisation of the epithelium and compromised epithelial integrity (Fig 3M–3N”). The basal surface of *dpix*, *git* tissues displayed intense accumulations of activated myosin at the center of aberrant multicellular junctions (arrowhead in Fig 3N”). We also saw purse-string like contractions of pMRLC at the center of cell rosettes that appeared to be ‘holes’, representing a breach of the continuity of the follicular epithelium (Fig 3N–3N”, and asterisk in Fig 3N”). Taken together these data indicate that loss of *dpix* and *git* results in dysregulated myosin activity, which causes cell deformation (compare Fig 2P and 2Q) and disruption to tissue integrity (Fig 3M and 3N).

### Reduction of myosin activators restores egg formation and morphology in the absence of *dpix* and *git*

**Fig 4:**
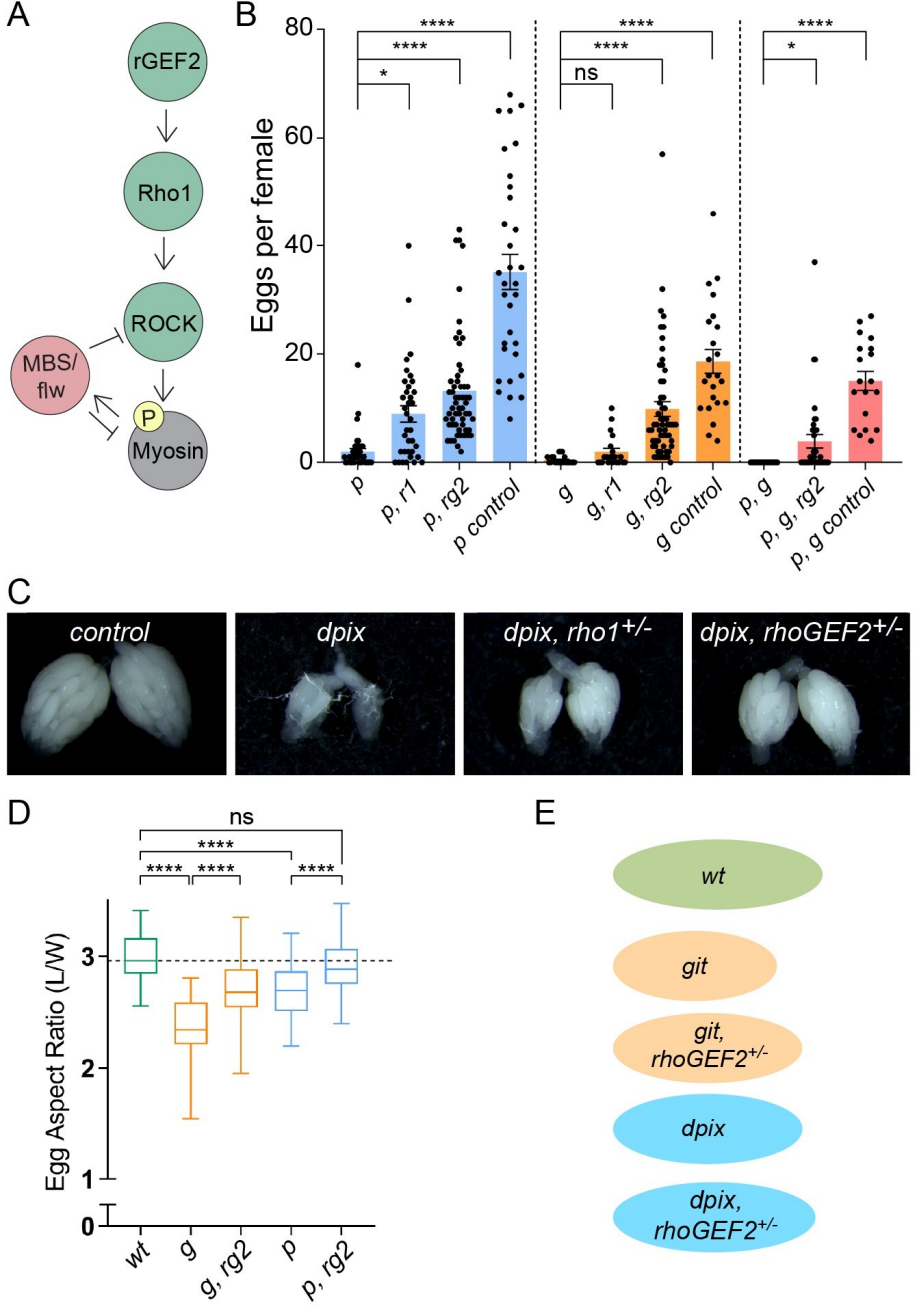
Reduction of myosin activators rescues egg chamber viability and tissue morphology defects in the absence of the dPix-Git complex. **(A)** Schematic of signalling cascade for non-muscle myosin regulatory light chain (Myosin) phosphorylation in *D*. *melanogaster*, highlighting the role of Rho1 and RhoGEF2 (rGEF2) as canonical upstream activators of myosin. **(B)** Number of mature eggs produced per female for the indicated genotypes. Statistical tests are ANOVA with post hoc Tukey’s test. Genotype groupings considered for statistical comparison are indicated by the dashed lines in (B), and error bars are standard error of the mean. Genotypes and sample sizes as follows: (*p*) *dpix*, n = 37; (*p*, *r1*) *dpix*, *rho1*^*+/-*^, n = 36; (*p*, *rg2*) *dpix*, *rhoGEF2*^*+/-*^, n = 57; (*p control*) *Df(2L)*^*+/-*^, n = 32; (*g*) *git*, n = 21; (*g*, *r1*) *git*, *rho1*^*+/-*^, n = 20; (*g*, *rg2*) *git*, *rhoGEF2*^*+/-*^, n = 57; (*g control*) *Df(2R)*^*+/-*^, n = 23; (*p*,*g*) *dpix*, *git*, n = 37; (*p*, *g*, *rg2*) *dpix*, *git*, *rhoGEF2*^*+/-*^, n = 36; (*p*, *g control*) *dpix*^*+/-*^, *git* ^*+/-*^, n = 19. Significance: * = p < 0.05; **** = p < 0.0001; ns = not significant. **(C)** Representative images of ovary development and egg production. Genotypes from left to right are: (control) *Df(2L)*^*+/-*^; *dpix*; *dpix*, *rho1*^*+/-*^; *dpix*, *rhoGEF2*^*+/-*^. **(D)** Reducing the gene dose of myosin activators increases mature egg length in *git* mutants and rescues egg length in *dpix* mutants. Upper and lower box edges represent upper and lower quartile boundaries, whiskers represent min to max. Genotypes and sample sizes are as follows: (*wt*) wild-type, n = 51; (*g*) *git*, n = 18; (*g*, *rg2*) *git*, *rhoGEF2*^*+/-*^, n = 73; (*p*) *dpix*, n = 108; (*p*, *rg2*) *dpix*, *rhoGEF2*^*+/-*^, n = 97. Significance: **** = p < 0.0001; ns = not significant. **(E)** Illustration of average aspect ratios for the indicated genotypes.

To test whether the ectopic myosin activation we detected could be a major cause of impaired egg chamber development, we set out to reduce the aberrant myosin activity in *dpix* and *git* mutants. To do this we generated animals that were homozygous mutant for *dpix* or *git*, and heterozygous for either of the canonical activators of myosin, *rhoGEF2* or *rho1* (Fig 4A). We selected these genes in line with the hypothesis that a Git-dPix-Pak signalling axis regulates egg chamber development; and following previous work showing that heterozygosity for *rho1* and *rhoGEF2* rescued egg production in *pak* mutants [22,49], and that RhoGEF2 regulates Rho1 which in turn regulates myosin in the basal region of egg chamber follicle cells [10,11]. Heterozygosity for *rhoGEF2* was sufficient to increase egg production in both *dpix* and *git* mutants (Fig 4B, with representative images for *dpix* in Fig 4C). Similarly, heterozygosity for *rho1* increased egg production in *dpix* animals but to a lesser degree than *rhoGEF2*. *rho1* heterozygosity also raised average egg production in *git* mutants but this increase was not statistically significant for the number of animals analysed (Fig 4B). As *rhoGEF2* heterozygosity conferred a robust increase of mature egg production in *dpix* and *git* mutants we engineered animals that were homozygous mutant for both *dpix* and *git*, and heterozygous for *rhoGEF2*. Strikingly, although *dpix*, *git* mutants never produced mature eggs, when we halved the gene dose of *rhoGEF2* egg production was often restored (Fig 4B).

While the major defect of *dpix* and *git* mutants is reduced egg chamber viability, we also noticed that surviving mature eggs had an elongation defect (Fig 4D and 4E and S3I and S3J Fig). Remarkably, heterozygosity for *rhoGEF2* significantly increased egg aspect ratio in *git* mutants, and generated an average aspect ratio of *dpix* mutant eggs comparable to wild-type (Fig 4D and 4E). Taken together, the increase in egg production, restoration of egg production in *dpix*, *git* mutants, and normalization of egg morphology indicate that aberrant myosin activation in *dpix* and *git* mutant animals has severe physiological consequences for egg chambers, and is one of the major defects in the absence of the dPix-Git complex.

### dPix and Git localise basally and to the leading edge of migrating cells

**Fig 5:**
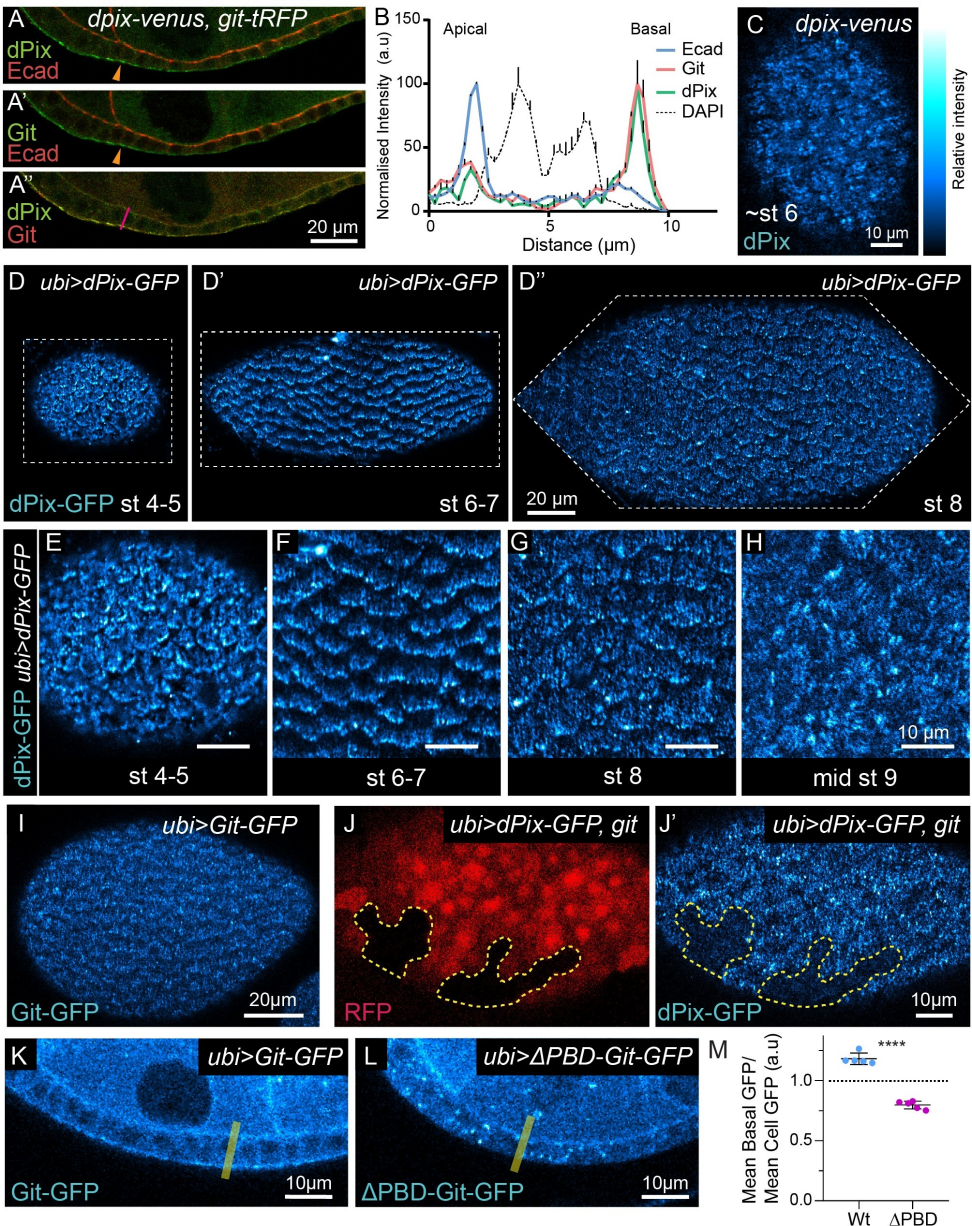
The dPix-Git complex is basally localised in follicular epithelia, and Git targets the dPix-Git complex to basal filaments and the leading edge via a paxillin targeting domain. **(A-A”)** Follicular epithelia expressing endogenously tagged alleles of *dpix* and *git*, bearing Venus and tRFP respectively, scale bar 20 μm. (A) dPix-Venus (dPix) costained with E-Cadherin (Ecad). (A’) Git-tRFP (Git) costained with Ecad. (A”) Co-localisation of dPix-Venus and Git-tRFP fluorescent signal. **(B)** Normalised fluorescence intensity profiles for dPix-Venus, Git-tRFP, Ecad and DAPI, from an apical-basal transect through the cell indicated by magenta line in (A”). Error bars show plus standard error of the mean. dPix-Venus and Git-tRFP signal were correlated with one another (Pearson’s correlation coefficient of 0.94, p < 0.0001), but did not correlate with Ecad (correlation coefficient of 0.21 and 0.2 for dPix-Venus and Git-tRFP respectively, and p > 0.05). **(C-C’)** Basal section of dPix-Venus expressing egg chamber demonstrating arrangement into parallel filaments. Image treated with Gaussian blur for noise reduction (σ = 0.5). Scale bar 10 μm. **(D-D”)** Basal localisation of ubiquitously expressed dPix-GFP transgene at the indicated stages of egg chamber development. Dashed lines indicate image boundaries. Scale bar 20 μm. **(E**-**G)** Enlargements of dPix-GFP from egg chambers in (D-D”), scale bars 10 μm. dPix-GFP at stage 4–5 (E), stage 6–7 (F), stage 8 (G). **(H)** Basal localisation of dPix-GFP during stage 9, after epithelial rotation has ceased. **(I)** Basal localised signal of ubiquitously expressed Git-GFP. Scale bar 20 μm. **(J-J’)** Localisation patterns of ubiquitously expressed dPix-GFP in epithelia that is a mosaic of wild-type and *git* mutant tissue. (J) *git* tissue is marked by the absence of RFP, and in (J-J’) is outlined by dashed yellow line. (J’) dPix-GFP localisation in *git* mutant tissue. Scale bar 10 μm. **(K**-**L)** Examples of Git-GFP (K) and ΔPBD-Git-GFP (L) localisation in follicular epithelium, with illustrative example of transect used for intensity quantification (yellow line). **(M)** Quantification of basal enrichment of GFP signal for Git-GFP (Wt) and ΔPBD-Git-GFP (ΔPBD). Statistical test is Student’s t-test, and error bars are standard deviation. Significance: **** = p < 0.0001. Sample sizes: Wt, n = 5; ΔPBD, n = 5. Relative intensity index applies to Venus and GFP signal in (C-L).

Having established an essential role for the dPix-Git complex in egg chamber development, and a central role in regulating epithelial monolayering, cell intercalation and myosin inhibition, we wanted to investigate the expression and localisation of these proteins during development. Therefore, we used CRISPR-Cas9 genome editing to add fluorescent tags to the 3’ end of the endogenous *dpix* and *git* open reading frames, creating *dpix-venus* and *git-tag Red Fluorescent Protein* (tRFP) transgenic animals, which were viable with no obvious defects. dPix-Venus and Git-tRFP were expressed in the germ line and follicular epithelium throughout ovary development with a basally enriched localisation in follicular epithelium (Fig 5A and 5B). We characterized this in stage 8 follicular epithelia, where dPix-Venus and Git-tRFP had a predominantly basal localisation (Fig 5A and 5B), but also accumulated to a lesser degree at apical membranes (Fig 5B). dPix-Venus and Git-tRFP appeared to co-localise and, consistent with this, the signal from these proteins co-varied (Fig 5B). Strikingly, on the basal side of the follicular epithelium dPix-Venus had a polarised localisation and was localised onto filament like structures, that extended perpendicular to the axis of egg chamber elongation (Fig 5C).

Our endogenously tagged *dpix* and *git* strains revealed a predominately basal localisation of this complex. However, endogenous expression levels produced very low fluorescent signals, and in an effort to better visualise localisation to subcellular structures we also generated animals expressing transgenes encoding C-terminal green fluorescent protein tagged dPix (*dPix-GFP*) and Git (*Git-GFP*), under the control of the *ubiquitin* promoter (*ubi*>) [55]. The *dpix* locus is annotated as encoding seven transcripts, and here we chose to clone a cDNA encoding RtGEF-A (dPix-A) as it contains all major conserved protein domains. To confirm that these transgenes are functional, and to avoid competition between dPix-GFP, Git-GFP and endogenous dPix and Git we recombined each of these transgenes into a corresponding mutant background.

Confocal imaging between stages 4 to 8 revealed a striking planar polarised localisation of dPix-GFP across the entire basal surface of the follicular epithelium (Fig 5D–5D”). This mirrored our observations with endogenously tagged dPix, but was more readily observable. We found two distinct polarised localisations of dPix-GFP. First, consistent with our observations for endogenous dPix-Venus, we saw that in each cell dPix-GFP localised along filament like structures that resembled basal actin filaments, perpendicular to the axis of elongation in each egg chamber (Fig 5E–5G). Second, during stages of collective cell migration, dPix-GFP was highly enriched towards one cell membrane and the direction of polarised localisation of dPix-GFP was shared between every cell in a single egg chamber (Fig 5E–5G). Further, dPix-GFP punctae were observed at the ends of dPix-GFP filament structures (Fig 5E–5G). To determine whether dPix-GFP localised towards the trailing or leading edge of these cells, we live imaged egg chamber rotation and found that dPix-GFP accumulated most strongly immediately behind the leading edge membrane of migrating follicle cells (S4A Fig). Notably, the planar polarised localisation of dPix-GFP was lost at stage 9 (Fig 5H) following the termination of collective migration. A similar pattern of localisation was seen for Git-GFP, however, enrichment at the leading edge was less pronounced (Fig 5I).

Given that dPix and Git often function in a complex and are able to influence each other’s localisation in cell culture [31,56], we assessed whether Git was responsible for targeting dPix to basal filaments and the leading edge. To test this we made clones of *git* in the context of *dPix-GFP* expression and found that loss of *git* prevented dPix-GFP localisation to both structures at the basal region of cells (Fig 5J–5J’). Having seen that Git controls the basal localisation of the dPix-Git complex we also wanted to know what determined Git localisation in this system. In mammals, GIT1/2 contain a C-terminal paxillin binding domain which targets GIT proteins to the focal adhesion protein paxillin, allowing for recruitment of PIX and PAK [25,31]. This paxillin binding domain appeared conserved in *D*. *melanogaster*, and so we removed the C-terminus of Git (*ΔPBD-Git-GFP*), and found that while Git-GFP accumulated in the basal region of cells, any basal GFP enrichment above average levels found throughout the cell was lost in ΔPBD-Git-GFP (Fig 5K–5M).

The basal and leading edge localisation of dPix and Git prompted us to look for defects in actin alignment which are indicative of defects in follicle cell migration [57]. We found incompletely penetrant defects in the global alignment of basal actin filaments in stage 8 *git* egg chambers (S4B and S4C Fig). However, live imaging showed conclusively that egg chamber rotation continues in *dpix* and *git* mutants (S4D–S4F Fig), and the speed of rotation is in fact enhanced in *git* mutants (S4F Fig). Collectively, these experiments reveal that dPix and Git are predominantly basally localised, and that Git’s paxillin binding domain targets the entire complex to basal filaments, and the leading edge of each migrating cell. They further suggest that Git plays a role in limiting the speed of follicle cell migration.

### Mechanism of action of dPix and Git in egg chamber development

**Fig 6:**
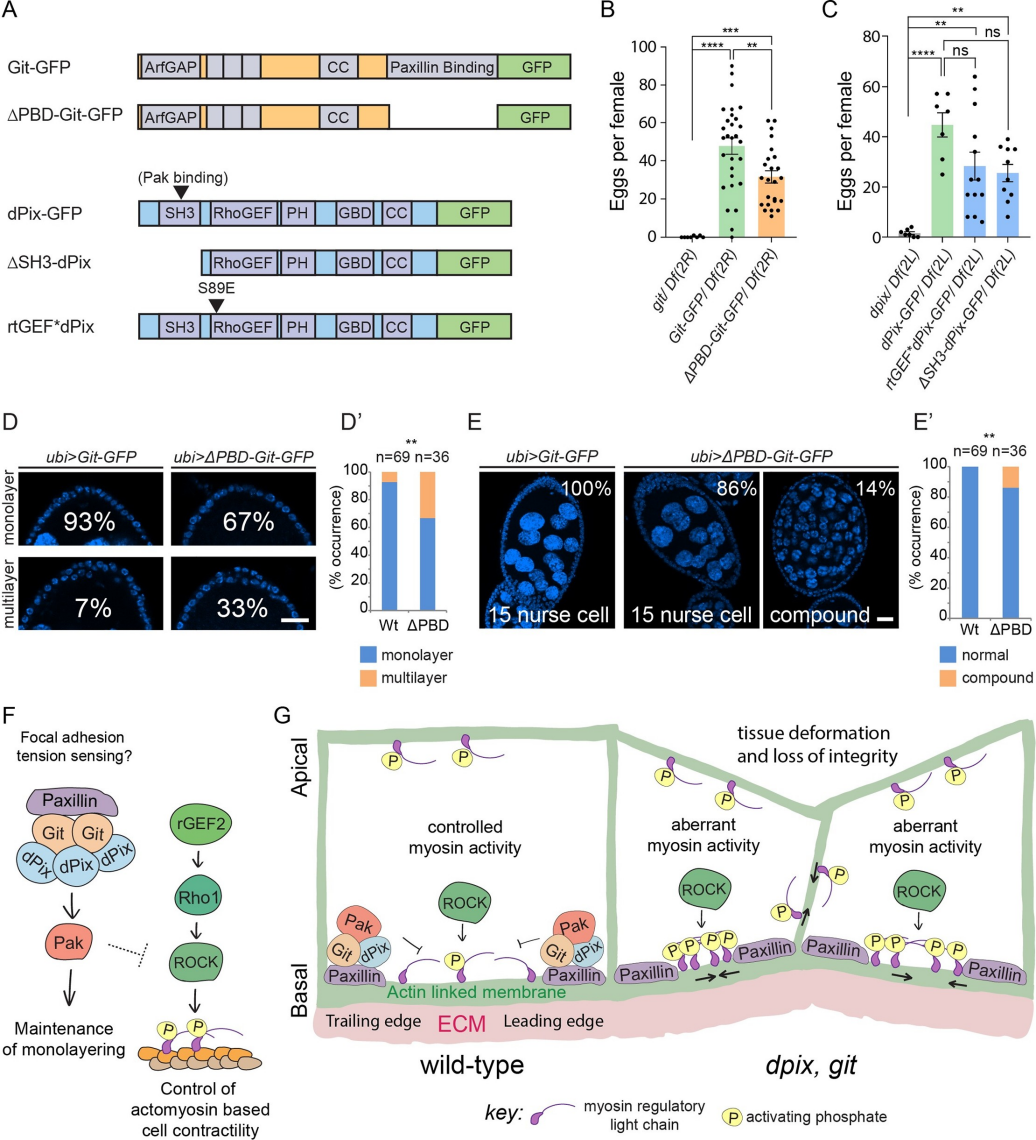
Focal adhesion localisation of the dPix-Git complex improves egg production and reduces morphogenetic defects. **(A)** Schematic of dPix and Git transgenic rescue and structure function constructs with key domains indicated. Abbreviated annotations are: Coiled-coil (CC), Pleckstrin homology (PH), and Git binding domain (GBD). **(B**-**C)** Number of mature eggs produced per female for the indicated genotypes. Statistical tests are ANOVA, with post hoc Tukey’s test, and error bars are standard error of the mean. (B) Genotypes and sample sizes are: *git/ Df(2R)*, n = 8; *Git-GFP/ Df(2R)*, n = 29; *ΔPBD-Git-GFP/ Df(2R)*, n = 24. (C) Genotypes and sample sizes are: *dpix/ Df(2L)*, n = 7, *dPix-GFP/ Df (2L)*, n = 7; rtGEF*dPix-GFP/ Df(2L), n = 13; *ΔSH3-dPix-GFP/ Df(2L)*, n = 10. Significance: ** = p < 0.01; *** = p < 0.001; **** = p < 0.0001; ns = not significant. **(D**-**D’)** Examples and frequencies of monolayered and multilayered follicular epithelia in *ubi>Git-GFP* (Wt in D’) and *ubi>ΔPBDGit-GFP* (ΔPBD in D’) rescue constructs. Scale bars are 20 μm. (D’) Stacked bar chart of frequencies in (D), statistical test is two-sided Fisher’s exact test. Significance: ** = p < 0.01. **(E**-**E’)** Examples and frequencies of normal and compound germline cysts in *ubi>Git-GFP* (Wt in E’) and *ubi>ΔPBD-Git-GFP* (ΔPBD in E’) rescue constructs. Scale bar is 20 μm. (E’) Stacked bar chart of frequencies in (E), statistical test is two-sided Fisher’s exact test. Significance: ** = p < 0.01. **(F)** Model for the basal signalling network controlling activation of myosin and monolayering during early egg chamber development. **(G)** Schematic of the effect of *dpix*, *git* mutation on epithelial organisation during egg chamber development. Abbreviated annotation: Extracellular matrix (ECM).

Given the central role of dPix-Git in egg chamber development, we wanted to know how this signalling module is activated and how it activates downstream effectors such as Pak. To begin to dissect the signalling mechanisms of this complex we compared the phenotypes of wild-type *dPix-GFP* and *Git-GFP* transgenes with a series of structure function alleles (Fig 6A) we had generated and incorporated into identical genomic locations via the phiC31 integrase to ensure even expression. In each case *dpix* and *git* rescue transgenes were expressed in the corresponding mutant background.

First, we confirmed that ubiquitously expressing a single copy of wild-type *dPix-GFP* or *Git-GFP* in the corresponding mutant background, produced healthy adult *D*. *melanogaster* and also increased egg production (Fig 6B and 6C). Next, to investigate whether localisation of dPix and Git to focal adhesions was functionally important for egg chamber development we used mature egg production as an assay (Fig 6B and 6C). We counted the number of mature eggs in females and found that although Git’s focal adhesion targeting domain was not essential for Git function in egg development, removing this domain (*ΔPBD-Git-GFP*) reduced the average number of mature eggs per female by about 30 percent (Fig 6B). To investigate signalling downstream of dPix and Git, we removed the dPix SH3 domain (*ΔSH3-dPix-GFP*), as the SH3 domain has been shown to mediate the interaction of PIX and PAK proteins [33,58]. *ΔSH3-dPix-GFP* expression in *dpix* mutant animals increased egg production to a similar level as full length dPix-GFP, suggesting that Pak binding via the SH3 domain is not essential for much of dPix function in egg chamber development (Fig 6C). Additionally, removing the dPix SH3 domain did not noticeably affect its localisation (S5A–S5E Fig, and compare to Fig 5D–5G). As dPix is a putative activator of Rac1 and Cdc42 GTPases via its RhoGEF domain, we also mutated a key serine residue that is conserved as either serine/threonine in RhoGEF domains, and is known to be required for activation of Rac1 in vitro [59]. Expression of a transgene encoding dPix where serine 89 was mutated to glutamic acid (rtGEF*dPix-GFP) rescued egg production in *dpix* mutants similar to full length *dPix-GFP* (Fig 6C), indicating that this conserved residue is not essential for dPix function in egg chamber development. Thus, although these experiments do not provide clarity on the molecular mechanism by which dPix functions in egg development they do suggest the possibility of redundant signalling mechanisms of dPix in this tissue.

To further investigate the requirement of focal adhesion targeting of Git in egg chambers, we compared the frequency of morphological defects in homozygous *Git-GFP* and *ΔPBD-Git-GFP* expressing *D*. *melanogaster*. We saw that removal of focal adhesion targeting resulted in a large increase in rates of multilayering (Fig 6D–6D’). We also found that expression of *ΔPBD-Git-GFP* led to compound egg chamber defects where developing egg chambers contained more than the normal complement of 15 germline nurse cells (Fig 6E–6E’). Thus loss of focal adhesion targeting increases the rate of defects normally seen in *git* homozygous mutants, implicating paxillin binding in the maintenance of monolayering and germline cyst encapsulation.

## Discussion

### Conclusions

Precise subcellular control of the contractile force-generating protein myosin has emerged as one of the most important determinants of tissue morphogenesis and organ development [60–63]. While much is known about apical regulators of myosin [64–67], less is known about the basal control of myosin activity in developing tissues [10–12]. Here we have identified an essential requirement for the basal dPix-Git complex in epithelial morphogenesis during egg chamber development, and revealed a major physiological role for this complex in the spatial control of myosin activation. The basal localisation of the dPix-Git complex relies on Git’s focal adhesion targeting domain, and loss of this targeting reduced egg production, suggesting that focal adhesions are a key site of dPix-Git activity. Additionally, the precise phenocopy of a side-by-side egg chamber fusion defect that to our knowledge has only been reported in *pak* mutants, suggest that a major part of dPix-Git function is mediated through Pak. Taken together our data indicate that the dPix-Git complex functions with Pak to respond to information sensed at focal adhesions during egg chamber development.

### An essential role of dPix-Git in the regulation of myosin

We have shown that loss of either *dpix* or *git* leads directly to aberrant myosin activation and that misregulated myosin is a major physiological cause of defective development of *dpix* and *git* egg chambers. Loss of *dpix* and *git* leads to force anisotropies at the basal surface of epithelia and collapsed lateral membranes. In *dpix* and *git* cells, myosin activation is likely to be a consequence of lowered Pak activation [22,49]. Thus, our results support the role of Pak in antagonising myosin during egg chamber development, and also identify a potential mechanism for both the basal localisation of Pak and spatial control of Pak activation. The regulation of myosin is critical to the morphogenesis of egg chambers. This protein of diverse functions requires different sets of regulators in order to be manipulated into an extraordinary range of emergent behaviours, often in different compartments of the very same cell. In early stage egg chambers myosin is required apically to resist the pressure of the growing germline cyst which would otherwise deform the follicular epithelium [14]. There is now also evidence that apical-medial myosin generates pulsatile contractions in the earliest stages of egg chamber elongation [15]. At the basal cell surface during the processes of collective cell migration, retrograde flow of myosin has been reported opposite to the direction of cell movement [13]. At the cessation of migration (stage 9), basal myosin switches from retrograde flow to patterns of asynchronous oscillations controlled by an integrin-ROCK cascade [10,11]. Given our identification of dPix and Git as key regulators of myosin inhibition in egg chambers, it will be important to determine which subsets of these dynamic processes dPix and Git control.

In addition to increasing mature egg production, we also found that halving the gene dose of myosin activators could rescue the aspect ratio of mature *dpix* and *git* eggs. Two potential causes for this short mature egg phenotype seen in *dpix* and *git* mutants are defects in nurse cell dumping, and defects in vitellogenesis [68]. Each of these phenotypes can also be explained by alterations in myosin contractility. In the case of nurse cell dumping, this defect would be in the germline nurse cells. In the case of vitellogenesis this defect would be in the muscle sheath which encloses developing egg chambers. Taken together this elongation data is consistent with a physiological role for *dpix* and *git* in regulating myosin in cells beyond the follicular epithelium.

Given our evidence that a dPix-Git-Pak module potently limits the activation of myosin, it is important to understand the molecular mechanism of myosin regulation downstream of this complex. Interestingly, while mammalian studies have shown that PAK1 can inactivate myosin by inhibiting myosin light-chain kinase (MLCK) [69], *D*. *melanogaster* MLCKs that mediate Pak’s role in egg chamber development have not been identified [22]. However loss of *pak* has been shown to lead to a redistribution of RhoGEF2 within follicle cells [22], highlighting misregulation of Pak and RhoGEF2 as a plausible mechanism for the deregulation of myosin in the absence of dPix and Git. The importance of targeting of dPix-Git to focal adhesions in egg chambers is also consistent with the hypersensitivity of integrin mutants to myosin overexpression [9]. In particular, the dPix-Git orthologues bind to the focal adhesion protein paxillin, and previous *D*. *melanogaster* studies have shown that overexpression of paxillin can suppress Rho-myosin induced phenotypes, and enhance Rac signalling phenotypes [70]. Thus, our data reinforces a model that some signalling cascades downstream of integrins and paxillin can suppress Rho activation during epithelial morphogenesis, and extend this model to suggest that dPix-Git binding to paxillin is a critical part of this mechanism.

### dPix-Git as key physiological activators of Pak in follicular epithelia

Cell biological and in vivo studies have identified PIX and GIT as regulators of PAK in a number of biological systems. Prior to this study, the regulators of Pak during egg chamber development were unknown. Here we report that *dpix* and *git* phenocopy loss of *pak* in egg chamber development in terms of multilayering, aberrant myosin activation, and in the generation of cell intercalation defects [49]. The biochemistry of PAK activation is well understood, however the in vivo cell biological basis of PAK activation by PIX-GIT proteins is less clear [71]. Two possibilities in egg chambers include that: dPix-Git activate Pak via the Rho family GTPases Rac1/Cdc42; and/or that dPix-Git promote local accumulation and autophosphorylation of Pak kinase. Using *ubiquitin* promoter-driven rescue constructs we found that removing the SH3 domain (Pak binding), or mutating the GEF domain of dPix still strongly increased egg development in *dpix* mutants. This raises the possibility of redundant mechanisms for Pak activation in this system. For instance, in the absence of an SH3 domain which mediates the dPix-Pak interaction, basal levels of activation of Rac or Cdc42 may still provide sufficient Pak activation. Similarly, in the absence of an efficiently functioning GEF domain, the dPix-Git complex may still serve as a basally located scaffold for accumulation and activation of Pak. Further clarity on dPix signalling in egg development could be provided by manipulating the endogenous *dpix* locus.

### A role of mechanical signalling through focal adhesions in egg chamber development

Mechanical forces can guide tissue development by passively constraining tissue shape, but also by the processes of mechanotransduction where forces applied to the cell surface are translated into biochemical signals that propagate inside the cell. Focal adhesions contain proteins which stretch and change molecular interactions under tension, and these molecules form the basis of much of our knowledge of mechanotransduction. The focal adhesion localised PIX, GIT and PAK module functions as a bona fide mechanotransducer in a range of systems. In *D*. *melanogaster*, Git is recruited to integrin-based adhesions in response to embryonic muscle contractions [72]. In *C*. *elegans*, the PIX-GIT-PAK orthologues respond downstream of cell attachments to promote intermediate filament phosphorylation and embryonic elongation [73]. In mammalian cell culture, PIX and GIT proteins are thought to act as mechanosensors due to their tension mediated interaction with paxillin at focal adhesions. For example, mechanical activation of focal adhesion kinase (FAK) via integrins and substrate stiffness [74,75], or by direct stretching of the FAK molecule [76] can lead to FAK’s interaction with SRC and the direct phosphorylation of GIT proteins [25,77]. GIT2 phosphorylation by FAK/SRC unmasks paxillin binding sites and localises the PIX-GIT-PAK complex to focal adhesions where it becomes a platform for signal propagation [77] (reviewed in [78]). Given that *D*. *melanogaster* egg chambers exhibit robust gradients of anisotropic extracellular matrix stiffness along the anterior-posterior axis [79], it will be interesting to further test whether dPix and Git act as mechanostransducers downstream of the stiffness gradient in this system.

### Localisation and function of dPix-Git at the leading edge of migrating epithelial cells

At present, few proteins have been identified that are specifically enriched at the leading edge of each follicle cell during collective migration, and the dPix-Git complex represents a new component of this domain. Although we found evidence that this targeting depends on Git, it is not yet clear how the dPix-Git complex becomes enriched at the leading edge. One possibility is that the molecular composition and phospho-signalling profile of newly formed focal adhesions at the leading edge of cells favours the interaction between dPix-Git and paxillin, relative to mature adhesions in the medial-basal region of cells. Alternatively, Fat2 cadherin is known to signal from the trailing edge of one cell, in order to organise the leading edge of the cell immediately ‘behind’ [80]. It will be interesting to see if Fat2 is required to organise dPix at the leading edge of cells in this system.

### The tissue specific requirement for the dPix-Git complex

A major finding of this study is that *dpix* and *git* are essential for epithelial morphogenesis in some tissues, but dispensable or act redundantly in others. We found that *dpix*, *git* mutation in *D*. *melanogaster* is semi-lethal, indicating that this complex is required in unidentified developmental contexts beyond egg chamber formation. Given this, it will be interesting to determine the role of dPix-Git in regulating myosin in other Pak*-*dependent developmental contexts, such as the epithelial sheet movement that drives dorsal closure in the embryo [81,82]. While cell culture studies have identified potential in vivo biological processes that the dPix-Git-Pak module regulates, our results underscore the essential role of animal studies to identify the precise situations where different protein complexes act in vivo. A key property of the integrin-dPix-Git-Pak signalling axis is that it can connect chemical and physical information from the ECM with intracellular signalling. This study and the work of others suggest a pattern where ECM sensing properties have made this module suitable for coordinating relatively specific developmental processes. These include control of cell migration [83,84], response to mechanical tension during epithelial morphogenesis [73], and the development of tube forming vascular systems (which require resistance to pressure) [23,85,86]. In the present case, the *D*. *melanogaster* follicular epithelium undergoes collective migration and must adjust to the pressure generated by an exponentially growing germline cyst [14,87]. Considering these animal studies together, our data suggest an underlying similarity between *D*. *melanogaster* egg chambers and tissues such as vertebrate vasculature. In the future it will be interesting and useful to transfer the understanding of how integrin-dPix-Git-Pak signalling guides development and maintains tissue integrity in these seemingly distant biological systems.

## Methods and materials

### *D*. *melanogaster* stocks and genetics

All *D*. *melanogaster* used in experiments were reared at 25°C unless otherwise indicated. For experiments assaying egg chamber development, adult female *D*. *melanogaster* were yeast fed in addition to a diet on standard medium, and were maintained in the presence of males. Unless otherwise indicated, homozygous *dpix* animals were generated by crossing the *dpix*^*p1036*^ allele to the *Df(2L)ED1315* deficiency, and homozygous *git* animals were generated by crossing the *git*^*ex21C*^ allele to the *Df(2R)BSC595* deficiency. For stock information, including stocks from the Bloomington *Drosophila* Stock Center (BDSC), and *Drosophila* Genomics and Genetics Resources (DGGR) stock center, see Table 1. For key information on the genotypes described in figures, see Table 2.

**Table 1:** Key resources.

***Drosophila melanogaster* stocks/ strains**			
Name	Source or Reference	Identifiers / Notes	Additional Information
*W1118*	BDSC	RRID:BDSC_3605	*w[1118]*
*dpix* ^*p1036*^	[34]		*w; dpix*^*p1036*^
*git*^*ex21C*^	[36]		*w; git*^*ex21C*^
*Df(2R)BSC595*	BDSC	RRID:BDSC_25428	*w[1118]; Df(2R)BSC595/CyO*
*Df(2L)ED1315*	BDSC	RRID:BDSC_9269	*w[1118]; Df(2L)ED1315*, *P{w[+mW*.*Scer\FRT*.*hs3] = 3'*.*RS5+3*.*3'}ED1315/SM6a*
*git*^*F03586*^	[35]		*w*; *git*^*F03586*^
*pak*^*6*^		RRID:BDSC_8809	*Pak[6]/TM3, Sb[1] Ser[1]*
*pak*^*11*^		RRID:BDSC_8810	*Pak[11]/TM3, Sb[1]*
*dpix*^*p1036*^ *FRT 40A*	[37]		*w; dpix*^*p1036*^ *FRT 40A*
*FRT 42D git*^*ex21C*^	[37]		*w; FRT 42D git*^*ex21C*^
*hsFLP; FRT 42D ubi-GFP*	Harvey Lab		*yw hsFLP; FRT 42D ubi-GFP*
*ubi-eGFP FRT40; T155-Gal4-UAS-FLP*	Horne-Badovinac Lab		*w; ubi-eGFP FRT40; T155-Gal4-UAS-FLP*
*Rho1[1B]*	BDSC	RRID:BDSC_9477	w[*]; b[1] pr[1] cn[1] Rho1[1B] px[1] sp[1]/CyO
*RhoGEF2[04291]*	BDSC	RRID:BDSC_11369	*cn[1] P{ry[+t7.2] = PZ}RhoGEF2[04291]/CyO; ry[506]*
*dpix-venus*	This study		*w*; *dpix-venus*
*git-tRFP*	This study		*w; git-tRFP*
*dpix-venus git-tRFP*	This study		*w; dpix-venus git-tRFP*
*ubi*:*dPix-GFP dpix* ^*p1036*^	This study		*w; ubi*:*dPix-GFP dpix* ^*p1036*^
*ubi*:rtGEF*dPix-GFP dpix ^*p1036*^	This study		*w; ubi*:rtGEF*dPix-GFP dpix ^*p1036*^
*ubi*:*ΔSH3-dPix-GFP dpix* ^*p1036*^	This study		*w; ubi*:*ΔSH3-dPix-GFP dpix* ^*p1036*^
*git*^*ex21C*^ *ubi*:*Git-GFP*	This study		*w; git*^*ex21C*^ *ubi*:*Git-GFP*
*git*^*ex21C*^ *ubi*:*ΔPBD-Git-GFP*	This study		*w; git*^*ex21C*^ *ubi*:*ΔPBD-Git-GFP*
*dpix*^*p1036*^ *git*^*ex21C*^	This study		*w; dpix*^*p1036*^ *git*^*ex21C*^
*dpix* ^*p1036*^ *git*^*F03586*^	This study		*w; dpix* ^*p1036*^ *git*^*F03586*^
*hsFLP; FRT42D ubi-RFP*	BDSC	RRID:BDSC_35496	*y[1] w[1118]; P{ry[+t7.2] = neoFRT}42D P{w[+mC] = Ubi-mRFP.nls}2R*
*Beta-heavy-spectrin-GFP*	DGGR	115258	Information available at DGGR database
*hsFLP; arm-lacZ FRT 40A*	Harvey Lab		*yw hsFLP; arm-lacZ FRT 40A*
**Primary Antibodies and Fluorophores**			
**Antibody**	**Source**	**Identifier**	**Dilution**
Rat Anti-*Drosophila* Cadherin, DE monoclonal antibody (Ecad)	DSHB	DSHB Cat# DCAD2, RRID:AB_528120	1:50
Mouse Anti-*Drosophila* fasciclin III, monoclonal antibody (FasIII)	DSHB	DSHB Cat# 7G10 anti-Fasciclin III, RRID:AB_528238	1:100
Mouse Anti-*Drosophila* Eyes absent antibody (Eya)	DSHB	DSHB Cat# eya10H6, RRID:AB_528232	1:100
Talin carboxy terminus 534 amino acids antibody (Talin)	DSHB	DSHB Cat# talin A22A, RRID:AB_10660289	1:50
Talin carboxy terminus 534 amino acids antibody (Talin)	DSHB	DSHB Cat# talin E16B, RRID:AB_10683995	1:50
Mouse Anti-*Drosophila* Orb monoclonal antibody (Orb)	DSHB	DSHB Cat# orb 4H8, RRID:AB_528418	1:30 (used with orb 6H4)
Mouse Anti-*Drosophila* Orb monoclonal antibody (Orb)	DSHB	DSHB Cat# orb 6H4, RRID:AB_528419	1:30 (used with orb 4H8)
Rabbit polyclonal Anti-Histone H3, phospho (Ser10)	Millipore	Millipore Cat# 06–570, RRID:AB_310177	1:250
Phospho-Myosin Light Chain 2 (Ser19) Antibody	Cell Signaling Technologies	Cell Signaling Technology Cat# #3671, RRID: AB_330248	1:20
Phalloidin-TRITC	Sigma	Sigma-Aldrich Cat# P1951, RRID:AB_2315148	1:200
CellMask Deep Red Plasma membrane Stain	Thermo Fisher Scientific	C10046	1:1000
**Chemicals peptide and recombinant proteins**			
Insulin solution from bovine pancreas	Sigma-Aldrich	I0516	
Schneider's *Drosophila *Medium	Thermo Fisher Scientific	21720024	
**Software**			
FIJI	[88]	https://fiji.sc/	
Ilastik	[89]	http://ilastik.org/	
CellProfiler	[90] Broad Institute	http://cellprofiler.org/	

**Table 2:** Experimental genotypes.

Figure#	Abbreviation in figure or legend	Key genotype information
**1, 2, 3, 4**	*wt*	*W1118*
**1, 2, 4, 6**	*dpix*	*dpix*^*p1036*^ */ Df(2L)ED1315*
**1, 2, 4, 6**	*git*	*git*^*ex21C*^ */ Df(2R)BSC595*
**1, 2, 3, 4**	*dpix*, *git*	*dpix* ^*p1036*^ *git*^*F03586*^ */ dpix* ^*p1036*^ *git*^*ex21C*^
**2**	*pak*^*6*^*/pak*^*11*^	*pak*^*6*^ */ pak*^*11*^
**2**	*pak*	*pak*^*6*^ */ pak*^*11*^
**1, 3**	*dpix* (mosaic)	*ubi-eGFP FRT40 / dpix* ^*p1036*^ *FRT 40A; T155-Gal4-UAS-FLP / +*
**1, 3**	*git* (mosaic)	*hsFLP / +; FRT 42D Ubi-GFP / FRT42D git* ^*ex21C*^
**4**	*dpix*, *rho1*^*+/-*^	*dpix* ^*p1036*^ *Rho1[1B] / Df(2L)ED1315*
**4**	*dpix*, *rhoGEF2*^*+/-*^	*dpix* ^*p1036*^ *RhoGEF2[04291] / Df(2L)ED1315*
**4**	*Df(2L)*^*+/-*^	*Df(2L)ED1315 / +*
**4**	*Df(2R)*^*+/-*^	*Df(2R)BSC595 / +*
**4**	*dpix*^*+/-*^, *git* ^*+/-*^	*dpix* ^*p1036*^ *git*^*ex21C*^ */ +*
**4**	*git*, *rho1*^*+/-*^	*git*^*ex21C*^ *Rho1[1B] / Df(2R)BSC595*
**4**	*git*, *rhoGEF2*^*+/-*^	*git*^*ex21C*^ *RhoGEF2[04291] / Df(2R)BSC595*
**4**	*dpix*, *git*, *rhoGEF2*^*+/-*^	*dpix* ^*p1036*^ *git*^*ex21C*^ *rhoGEF2[04291] / dpix* ^*p1036*^ *git*^*F03586*^
**5**	*dpix-venus*, *git-tRFP*	*dpix-venus git-tRFP*
**5**	*dpix-venus*	*dpix-venus*
**5**	*ubi>dPix-GFP*	*ubi*:*dPix-GFP dpix* ^*p1036*^
**5, 6**	*ubi>Git-GFP*	*git*^*ex21C*^ *ubi*:*Git-GFP*
**5**	*ubi>dPix-GFP*, *git*	*hsFLP / +; FRT 42D ubi-RFP / ubi*:*dPix-GFP dpix* ^*p1036*^ *FRT 42D git*^*ex21C*^
**5, 6**	*ubi>ΔPBD-Git-GFP*	*git*^*ex21C*^ *ubi*:*ΔPBD-Git-GFP*
**1, 6**	*git/ Df(2R)*	*git*^*ex21C*^ */ Df(2R)BSC595*
**1, 6**	*dpix/ Df(2L)*	*dpix*^*p1036*^ */ Df(2L)ED1315*
**6**	*Git-GFP/ Df(2R)*	*git*^*ex21C*^ *ubi*:*Git-GFP / Df(2R)BSC595*
**6**	*ΔPBD-Git-GFP/ Df(2R)*	*git*^*ex21C*^ *ubi*:*ΔPBD-Git-GFP / Df(2R)BSC595*
**6**	*dPix-GFP/ Df(2L)*	*ubi*:*dPix-GFP dpix* ^*p1036*^ */ Df(2L)ED1315*
**6**	rtGEF*dPix-GFP/ Df(2L)	*ubi*:rtGEF*dPix-GFP dpix ^*p1036*^*/ Df(2L)ED1315*
**6**	*ΔSH3-dPix-GFP/ Df(2L)*	*ubi*:*ΔSH3-dPix-GFP dpix* ^*p1036*^ */ Df(2L)ED1315*

### Immunohistochemistry

Ovaries were dissected in phosphate buffered saline (PBS) and fixed in 4% paraformaldehyde for between 10 and 15 minutes. Tissues were rinsed three times in PBS solution with 0.1% (v/v) Triton-X (PBS-T), and permeabilised for 20 minutes in PBS-T. Primary and secondary antibody incubations were overnight at 4°C in PBS-T with 10% (v/v) Normal Goat Serum (Sigma). Ovaries were washed 3 times for 10 minutes each in PBS-T following each antibody staining. DAPI staining was incorporated into the penultimate wash. Phalloidin stainings were for 1 hour at room temperature or overnight at 4°C when co-staining with antibodies. Stained tissues were stored and mounted in 90% glycerol with 10% PBS (v/v). Primary antibody and dye stains, including antibodies from the Developmental Studies Hybridoma Bank (DSHB), are as indicated in Table 1.

### Generation of endogenously tagged *dpix* and *git D*. *melanogaster* strains

The *dpix-venus* and *git-tRFP* strains were created via CRISPR/Cas-9 targeted transgene integration [91]. The sequence encoding either Venus fluorescent protein or tag Red Fluorescent Protein (tRFP) was inserted immediately 3’ of the stop codon of *D*. *melanogaster dpix* and *git* respectively, creating C-terminal fusion proteins. The donor vector contained approximately 1kb of homology on either side of a “knock in” cassette, which included the coding sequence for either Venus or tRFP, and a 3xP3-RFP [92] flanked by loxP sites. The gRNA expression vector used a 20-bp protospacer sequence, designed to include the *dpix* and *git* stop codons. The donor and gRNA vectors were each injected into fertilised eggs laid by nos-Cas9 flies [93]. Transformants were identified by eye specific red fluorescence from the 3xP3-RFP transgene, and this construct was then removed by crossing to a strain of *D*. *melanogaster* bearing a hs-Cre construct.

### Egg chamber rotation imaging, time lapse video acquisition and migration rate calculation

Experimental females were collected 1–3 days after eclosion and aged on yeast for 2 days in the presence of males. Ovaries were dissected in live imaging media (Schneider’s *Drosophila* medium with 15% FBS and 200 μg/ml insulin) containing either CellMask Deep Red or CellMask Orange Plasma Membrane Stain (Thermo-Fisher; 1:1000). Individual ovarioles were removed from muscle sheathes with forceps, transferred to fresh live imaging media, and then transferred to a glass slide. 51 μm Soda Lime Glass beads (Cospheric LLC) were added to support a 22 x 22 μm coverslip and Vaseline was used to seal the coverslip edges. Migration rates were determined for Stage 6 and Stage 7 egg chambers with the following exceptions: egg chambers with major structural defects including stalks fused along the follicle cell surface or multiple germ cell cysts within one egg chamber were excluded from analysis, as were damaged egg chambers as indicated by CellMask uptake. Egg chambers that exhibited follicle cell multilayering were included. Egg chambers were imaged with a Zeiss LSM 800 with a 40x/1.3 NA EC Plan-NEOFLUAR objective and Zen 2.3 acquisition software. Frames of a single plane near the basal epithelial surface were captured every 30 seconds for 20 minutes. To calculate epithelial migration rates, kymographs were generated from these movies in FIJI (ImageJ) by drawing a line across the egg chamber parallel to the migration path. The migration rate for each epithelium was then determined by measuring the slope of 4 kymograph lines and taking the mean of these values. This technique is illustrated in Barlan *et al*. 2017 [80].

Live imaging of ovary rotation to determine localisation of dPix-GFP at the leading or trailing edge followed the protocol in [94], and differed from the above as follows. Stained egg chambers were transferred to a gas permeable membrane (Ibidi) and imaged by encircling with petroleum jelly to serve as a spacer, with a glass coverslip placed on top. Images were acquired on a Nikon upright laser scanning confocal at intervals of 30 seconds, for approximately 20 minutes. To determine the localisation of dPix-GFP at the leading or trailing edge of cells, dPix-GFP and cell membranes were imaged at time zero. After finding the relative position of dPix-GFP accumulation and membranes, migration of membranes was imaged. dPix-GFP was not imaged as the signal from this construct photo-bleached rapidly.

### Generation of *dpix* and *git* rescue constructs

*dpix* and *git* rescue and structure function constructs were all generated by using Gateway cloning to introduce a sequence of interest into a derivative of the pKC26w-pUbiq rescue plasmid [55] (courtesy of Nic Tapon), containing a C-terminal green fluorescent protein tag (GFP). This plasmid provides ubiquitous expression under the control of the *ubiquitin-63E* promoter (denoted as “*ubi>*” in figures, and “*ubi*:” in key resources Table 1). This plasmid contains a mini-white coding sequence as a selectable marker. Constructs were inserted into *Drosophila* bearing *attP* landing sites via PhiC31 integrase-mediated transgenesis.

All *dpix* transgenes were inserted into the same chromosomal location on 2L, at site name VK37 (cytogenetic position 22A3) (BDSC 9752). All *git* transgenes were inserted into the same chromosomal location on 2R, at site name VK22 (cytogenetic position 57F5) (BDSC 9740). Injections were performed at BestGene.

As a wild-type *dpix* construct, we used coding sequence corresponding to *dpix* isoform A (http://flybase.org/reports/FBtr0081356) [37,95] (originally courtesy of Ed Manser), to create a construct that deletes the SH3 domain of dPix (aa 9–56) we designed primers to amplify a sequence that removes the first 59 amino acids of dPix. To remove RhoGEF activity from dPix we used site directed mutagenesis to convert serine 89 to glutamic acid [37,59]. To generate a *git* construct that does not bind to paxillin we designed primers to remove nucleotides encoding the last 125 amino acids of Git.

After creation of transgenic animals these *dpix* and *git* constructs were recombined onto chromosomes bearing *dpix* or *git* mutations, so that the transgenic construct was the only source of *dpix* or *git* in the genome. Recombinant offspring positive for the transgene were scored by the mini-white selectable marker and GFP expression, whereas mutation of endogenous *dpix* or *git* was scored by PCR of the relevant locus.

### Myosin intensity and PHH3 quantification

For image acquisition of pMRLC and E-cadherin in *dpix*, *git* mutants, stained ovaries were imaged on a Nikon laser scanning confocal. Staining was E-cadherin detected by anti-Rat-647, and pMRLC detected by anti-Rabbit-488.

Measurements of pMRLC intensity were performed in FIJI. Plots of intensity were generated using the “ggplot2” package in the R programming language. Frequency polygons were generated in GraphPad Prism 8 and used a bin width set to 0.2. For quantification of pMRLC and E-cadherin in *dpix*, *git* mutants, egg chambers from approximately stages 6–7 (before flattening of anterior follicular epithelium) were selected. Average apical and basal pMRLC and E-cadherin signal for an egg chamber was measured by selecting apical and basal regions from at least 19 cells per egg chamber, from regions that had maintained monolayering. pMRLC and E-cadherin signal were measured from the same cells and regions of interest for each egg chamber. Statistical tests were Welch’s two-sample t-test, performed in the R programming language.

To quantify follicle cell proliferation, we imaged z-stacks through egg chambers and the number of PHH3 positive cells were counted. Egg chamber staging used DAPI based features [50]. Boxplots for pMRLC intensity, E-cadherin intensity, and number of PHH3 positive cells, were generated using the “geom_boxplot” function in the “ggplot2” package of the R programming language.

### Venus, tRFP and GFP transgene imaging, and intensity quantification along transects

Measurements of transgene expression intensity in follicle cells were performed using FIJI software and GraphPad Prism 8. For Venus and tRFP transgenes, signal intensity was measured along three transects within the same cell. Intensity values for each channel were min-max normalised in GraphPad Prism 8. Pearson’s correlation coefficients were calculated using GraphPad Prism 8, and were obtained by first computing the mean intensity for each channel, and then analysing those means. For basally localised dPix-Venus (Fig 5C), the image was treated with Gaussian blur for noise reduction (σ = 0.5). For comparisons of basal localisation between Git-GFP and ΔPBD-Git-GFP, and dPix-GFP and ΔSH3-dPix-GFP, the apical membrane, cytoplasmic and basal membrane regions of each egg chamber were identified and labelled. Next GFP intensity was measured along a 10 pixel wide transect from the apical to basal region of individual cells. The average basal membrane GFP intensity was calculated for each cell, and normalised to the average total GFP intensity for the same cell. These measurements were made for at least three cells per egg chamber, and averaged to give an overall basal GFP enrichment value. Statistical tests to compare basal GFP enrichment between genotypes were Student’s t-test and were performed in GraphPad Prism 8.

### Generation of clonal mutant tissue

For *dpix* mosaic egg chambers, *dpix FRT40A D*. *melanogaster* were crossed to a *ubi-eGFP FRT40A; T155-gal4*, *UAS-FLP* strain, except for (S1H Fig) which used *hsFLP*, *arm-lacZ FRT40A*. The *hsFLP* system was used for *git* mosaic egg chambers. Clones were induced by heat shocking at 37 degrees Celsius for two hours, on two consecutive days, beginning when *D*. *melanogaster* had developed to 3^rd^ instar wandering larvae. Female offspring from these crosses were yeast fed for 3 days before dissection of egg chambers.

### Egg maturation rescue assays

To measure the effect of *rho1* and *rhoGef2* heterozygosity on egg production in *dpix* and *git* mutants, females were yeast fed for 3 to 5 days before dissection, at which point the number of stage 14 eggs were counted. For *dpix* and *git* transgene rescue experiments, females were allowed to mature on yeast for at least 3 and 5 days respectively, and stage 14 eggs were counted. Stage 14 eggs were scored by the presence of elongated dorsal filaments.

### Egg length and width measurement

Relative length and width of mature eggs between genotypes were measured by dissecting egg chambers into PBS and imaging with an Infinity camera mounted to a dissecting microscope. Length, width, and aspect ratio for mature eggs were determined in FIJI by measuring the ratio between the length of the longest and widest sections of each egg. Developing egg chambers were staged using DAPI based features [50]. In developing egg chambers length was measured along the anterior-posterior (AP) axis, and width was measured from the widest section perpendicular to the AP axis. Statistical tests were ANOVA, and were performed in GraphPad Prism 8. Boxplots of aspect ratio for developing egg chambers were generated using the “geom_boxplot” function in the “ggplot2” package of the R programming language.

### Cell morphological feature analysis

To measure cell morphological features of wild-type, *dpix* and *git* mutant egg chambers between stages 7 and 8, specimens were stained with phalloidin and the basal surface of follicle cells were imaged. We imaged main body follicle cells and avoided imaging cells at the egg chamber poles. Optical sections were segmented in Ilastik and exported to FIJI. Segmentation errors were manually corrected in FIJI and the Voronoi function was used to produce a skeletonized map of cell areas for object identification and measurement in CellProfiler. Cells at the edge of egg chambers were excluded from segmentation maps and were not measured. In CellProfiler the “IdentifyPrimaryObjects” module was used to identify cells, and the “MeasureObjectSizeShape” module was used to measure eccentricity. Statistical analysis was ANOVA with post hoc Dunnett’s test, performed in GraphPad Prism 8.

### Statistics

Significance values throughout are indicated as: * = p < 0.05; ** = p < 0.01; *** = p < 0.001; **** = p < 0.0001; ns = not significant. Numerical values underlying charts in the figures are provided (S1 Table).

## Supporting information

S1 Fig Characterisation of *dpix* and *git* mutant phenotypes. **(A)** General appearance of adult female *Drosophila melanogaster* of the indicated genotypes. From left to right: wild-type (*wt*); *git*; *dpix*; *dpix*, *git*. **(B**-**B”)** Example of multilayering (arrowheads in B”) in early stage *dpix* mutant egg chambers. Tissue morphology is indicated by E-cadherin (Ecad) (orange) and DAPI (blue) stains. Scale bar 20μm. **(C**-**D)** Quantification of relative Ecad intensity between inner (germline contacting) layer and outer (ectopic) layers of follicular epithelia for *git* (C) and *dpix* (D) homozygous mutants. Statistical tests are pairwise Student’s t-test. Sample sizes are: *git*, n = 11*; dpix*, n = 13. Significance: *** = p < 0.001; **** = p < 0.0001. **(E**-**E’)** Talin intensity (grey) in clones of *git* mutant tissue compared to adjacent *wt* tissue (green in E). The yellow line indicates the transect plotted in (F). Scale bar 40μm. **(F)** A plot of relative Talin signal along a transect of tissue (yellow line in E), with *git*, *wt* and *oocyte* regions indicated. **(G)** Example of cell autonomous induction of multilayering in *dpix* mutant clone in a stage 5 egg chamber. **(H**-**H’)** Comparison of β-heavy-spectrin-GFP (grey) localisation between wild-type cells (green in H) and *dpix* mutant cells (absence of green in H). Green colorization in (H) is Lac-Z staining as a marker of genotype. The dashed orange lines in (H-H’) indicate genotype boundaries. Scale bar 20μm. Genotype in (H-H’): *hsFLP / +; arm-lacZ FRT 40A / dpix*^*p1036*^ *FRT 40A; Beta-heavy-spectrin-GFP / +*.
(TIF)

S2 Fig Characterisation of proliferation in *dpix* and *git* mutants. **(A)** Box plot representation of quantification of phospho-Histone H3 staining (PHH3) positive cells in egg chambers of indicated genotypes and stages. Statistical tests are ANOVA. For stages 3–4: wild-type (*wt*), n = 10*; git*, n = 16*; dpix*, n = 11. For stages 5–6: *wt*, n = 12*; git*, n = 15*; dpix*, n = 19. For stages 7–9: *wt*, n = 15*; git*, n = 17*; dpix*, n = 23. Significance: ns = not significant. **(B)** Result of Fisher’s exact test for count data to test whether PHH3 status is independent of genotype between stages 7–9. **(C-D)** Quantification of relative Eyes Absent (Eya) intensity between inner (germline contacting) layer and outer (ectopic) layers of follicular epithelia for *git* (C) and *dpix* (D) homozygous mutants. Statistical tests are pairwise Student’s t-tests. Sample sizes are: *git*, n = 11*; dpix*, n = 9. Significance: *** = p < 0.001. **(E**-**G”)** Eya (orange) and DAPI (blue) stain, in the posterior region of single layered wild-type (E-E”), and multilayered *git* (F-F”) and *dpix* (G-G”) egg chambers between stages 8 and 10 as indicated. Scale bars 20μm. **(H**-**J”)** Examples of FasIII (orange) and DAPI (blue) stain, in the posterior region of single layered wild-type (H-H”), and multilayered *git* (I-I”) and *dpix* (J-J”) egg chambers. Scale bars 20μm.
(TIF)

S3 Fig Egg chamber and mature egg morphology defects in *dpix* and *git* mutants. **(A**-**C)** Examples of interfollicular stalks from wild-type (*wt*) (A), *git* (B) and *dpix* (C) homozygous mutant egg chambers visualised with DAPI (blue), showing examples of one cell wide stalk (arrow in A) and intercalation defects producing widened stalks in *git* and *dpix* mutants (orange arrow in B and C). Scale bars 20μm. **(D**-**E)** Examples of *git* (D) and *dpix* (E) compound egg chambers containing more than the wild-type complement of 15 nurse cells. Egg chambers are visualised with DAPI (blue) and nurse cells visible in the projected focal plane are indicated (yellow asterisk). **(F)** Quantification of relative frequency of compound and side-by-side fusion egg chambers for the indicated genotypes. Sample sizes are: wild-type (*wt*), n = 80*; git*, n = 108*; dpix*, n = 97. **(G**-**H”)** Examples of *dpix* (G-G”) and *git* (H-H”) fused egg chambers visualised with DAPI (blue) with oocytes marked by Orb staining (green in G’-G” and H’-H”). The presence of Orb staining within each set of enclosed germline cells indicates that fusions in *dpix* and *git* egg chambers are comprised of two germline cysts fused side-by-side. **(I**-**J)** Relative length (I) and width (J) of mature eggs for the indicated genotypes. In (I) and (J) sample sizes are: *wt*, n = 20; *dpix*, n = 33; *git*, n = 13. Statistical tests are ANOVA with post hoc Tukey’s test, and error bars are standard deviation. Significance: **** = p < 0.0001; ns = not significant.
(TIF)

S4 Fig dPix-GFP localises to the leading edge, and egg chamber rotation is enhanced in *git* mutants. **(A)** Kymograph from *ubi>dPix-GFP* expressing egg chamber showing the direction of cell migration in relation to polarised dPix-GFP localisation. The location of polarised dPix-GFP (green arrow) relative to cell membrane (red arrow) was determined at time zero. Live imaging of the membrane of migrating cells shows the direction of movement of the leading edge membrane, and indicates that polarised dPix-GFP is positioned immediately behind the leading edge membrane. Scale bar 1 μm. **(B**-**C)** Basal actin alignment (B) is disrupted with incomplete penetrance in *git* (C) follicle cells. **(D)** Representative example of follicle cell migration (cells tracked are marked in blue) over a 20 minute period in control (*+ / Df(2R)*), compared to *git* follicle cells. **(E)** Representative example of follicle cell migration (cells tracked are marked in blue) over a 20 minute period in control (*+ / Df(2L)*), compared to *dpix* follicle cells. **(F)** Quantification of migration rates in *git* and *dpix* mutants. Statistical tests are ANOVA with post hoc Tukey’s test. Genotypes and sample sizes are: + / *Df(2R)*, n = 24; *git/ Df(2R)*, n = 17; + / *Df(2L)*, n = 18; *Df(2L)/ dpix*, n = 13. Error bars are standard deviation. Significance: **** = p < 0.0001; ns = not significant. *D*. *melanogaster* deficiency stocks used for control and experimental genotypes in (D-F) are: *Df(2R)* = *Df(2R)BSC595*; *Df(2L)* = *Df(2L)ED1315*.
(TIF)

S5 Fig Subcellular localisation of dPix-GFP transgenes. **(A**-**B)** Examples of dPix-GFP (A) and ΔSH3-dPix-GFP (B) localisation in follicular epithelium, with illustrative example of transect used for intensity quantification (yellow line). Scale bars 10 μm. Colour key indicates relative intensity of GFP signal. **(C)** Quantification of basal enrichment of GFP signal in dPix-GFP (wt) and ΔSH3-dPix-GFP (sh3). Statistical test is Student’s t-test, error bars are standard deviation. n = 3 egg chambers per genotype. Significance: ns = not significant. **(D**-**E)** Examples of basal planar polarised enrichment of ΔSH3-dPix-GFP protein at the indicated egg chamber stages. Scale bars are 10 μm. Genotypes are: *ubi>dPix-GFP = ubi*:*dPix-GFP dpix* ^*p1036*^ (A, C); *ubi>ΔSH3-dPix-GFP = ubi*:*ΔSH3-dPix-GFP dpix* ^*p1036*^ (B-E).
(TIF)

S1 Table Underlying numerical data. Tables of the numerical data used to plot charts and perform statistical analysis of quantitative data.
(XLSX)
