# Supplementary figures and images for: The dPix-Git complex is essential to coordinate epithelial morphogenesis and regulate myosin during Drosophila egg chamber development
Source: PLoS Genet. 2019 May 22;15(5):e1008083. doi: 10.1371/journal.pgen.1008083 (PMC6555532; doi:10.1371/journal.pgen.1008083)

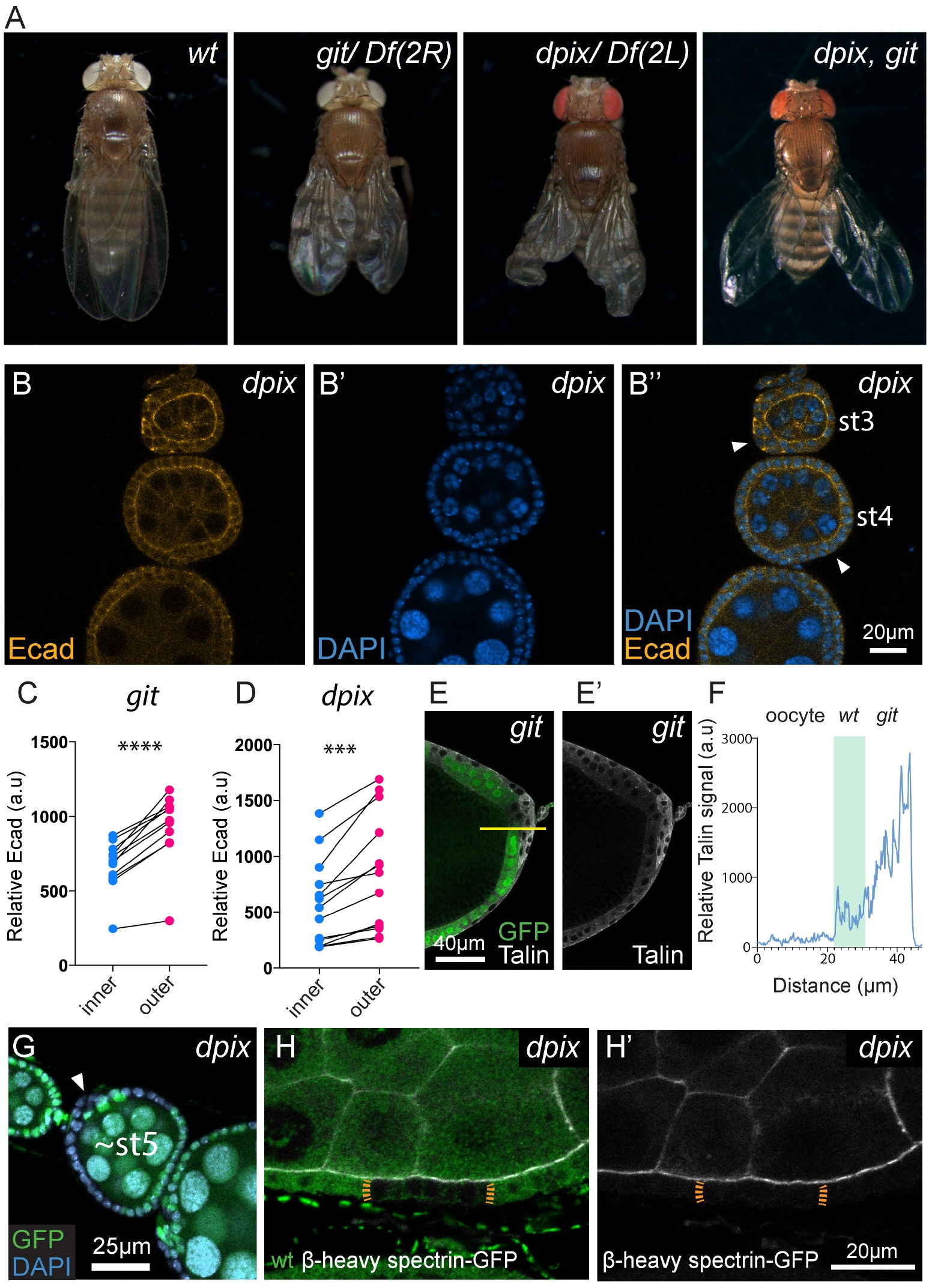

Supplement: S1 Fig — (A) General appearance of adult female Drosophila melanogaster of the indicated genotypes. From left to right: wild-type (wt); git; dpix; dpix, git. (B-B”) Example of multilayering (arrowheads in B”) in early stage dpix mutant egg chambers. Tissue morphology is indicated by E-cadherin (Ecad) (orange) and DAPI (blue) stains. Scale bar 20μm. (C-D) Quantification of relative Ecad intensity between inner (germline contacting) layer and outer (ectopic) layers of follicular epithelia for git (C) and dpix (D) homozygous mutants. Statistical tests are pairwise Student’s t-test. Sample sizes are: git, n = 11; dpix, n = 13. Significance: *** = p < 0.001; **** = p < 0.0001. (E-E’) Talin intensity (grey) in clones of git mutant tissue compared to adjacent wt tissue (green in E). The yellow line indicates the transect plotted in (F). Scale bar 40μm. (F) A plot of relative Talin signal along a transect of tissue (yellow line in E), with git, wt and oocyte regions indicated. (G) Example of cell autonomous induction of multilayering in dpix mutant clone in a stage 5 egg chamber. (H-H’) Comparison of β-heavy-spectrin-GFP (grey) localisation between wild-type cells (green in H) and dpix mutant cells (absence of green in H). Green colorization in (H) is Lac-Z staining as a marker of genotype. The dashed orange lines in (H-H’) indicate genotype boundaries. Scale bar 20μm. Genotype in (H-H’): hsFLP / +; arm-lacZ FRT 40A / dpixp1036 FRT 40A; Beta-heavy-spectrin-GFP / +. (TIF) [file pgen.1008083.s001.tif]

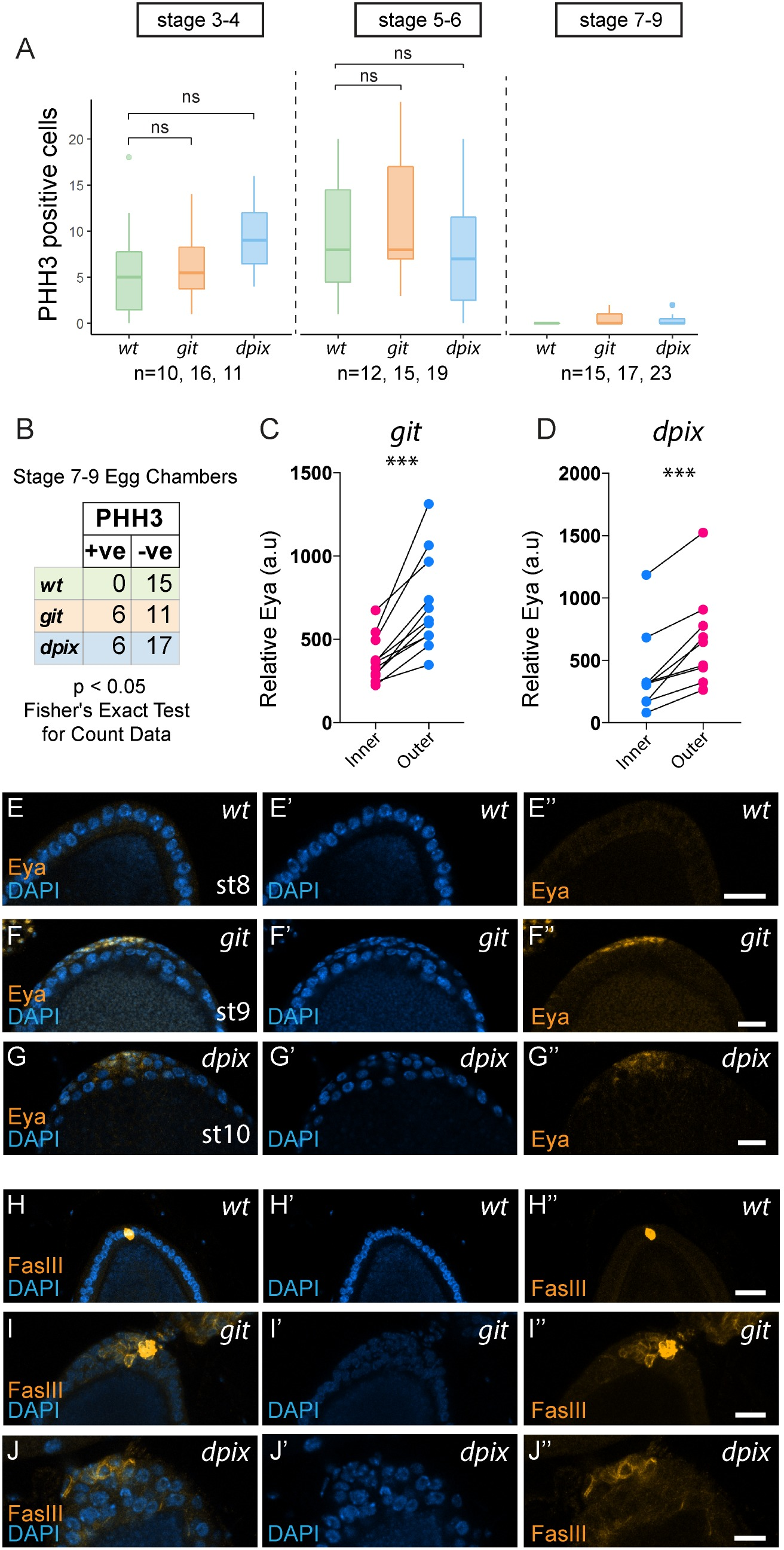

Supplement: S2 Fig — (A) Box plot representation of quantification of phospho-Histone H3 staining (PHH3) positive cells in egg chambers of indicated genotypes and stages. Statistical tests are ANOVA. For stages 3–4: wild-type (wt), n = 10; git, n = 16; dpix, n = 11. For stages 5–6: wt, n = 12; git, n = 15; dpix, n = 19. For stages 7–9: wt, n = 15; git, n = 17; dpix, n = 23. Significance: ns = not significant. (B) Result of Fisher’s exact test for count data to test whether PHH3 status is independent of genotype between stages 7–9. (C-D) Quantification of relative Eyes Absent (Eya) intensity between inner (germline contacting) layer and outer (ectopic) layers of follicular epithelia for git (C) and dpix (D) homozygous mutants. Statistical tests are pairwise Student’s t-tests. Sample sizes are: git, n = 11; dpix, n = 9. Significance: *** = p < 0.001. (E-G”) Eya (orange) and DAPI (blue) stain, in the posterior region of single layered wild-type (E-E”), and multilayered git (F-F”) and dpix (G-G”) egg chambers between stages 8 and 10 as indicated. Scale bars 20μm. (H-J”) Examples of FasIII (orange) and DAPI (blue) stain, in the posterior region of single layered wild-type (H-H”), and multilayered git (I-I”) and dpix (J-J”) egg chambers. Scale bars 20μm. (TIF) [file pgen.1008083.s002.tif]

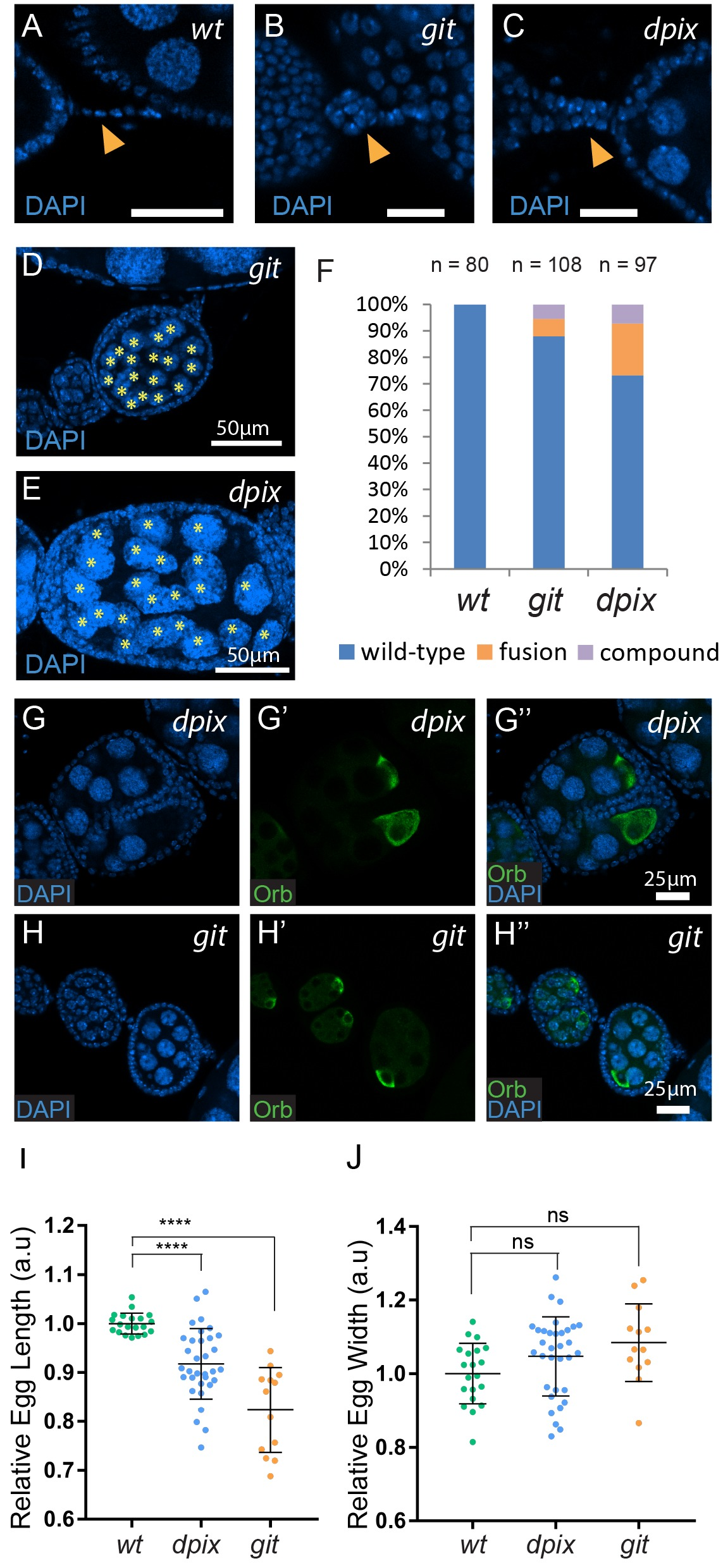

Supplement: S3 Fig — (A-C) Examples of interfollicular stalks from wild-type (wt) (A), git (B) and dpix (C) homozygous mutant egg chambers visualised with DAPI (blue), showing examples of one cell wide stalk (arrow in A) and intercalation defects producing widened stalks in git and dpix mutants (orange arrow in B and C). Scale bars 20μm. (D-E) Examples of git (D) and dpix (E) compound egg chambers containing more than the wild-type complement of 15 nurse cells. Egg chambers are visualised with DAPI (blue) and nurse cells visible in the projected focal plane are indicated (yellow asterisk). (F) Quantification of relative frequency of compound and side-by-side fusion egg chambers for the indicated genotypes. Sample sizes are: wild-type (wt), n = 80; git, n = 108; dpix, n = 97. (G-H”) Examples of dpix (G-G”) and git (H-H”) fused egg chambers visualised with DAPI (blue) with oocytes marked by Orb staining (green in G’-G” and H’-H”). The presence of Orb staining within each set of enclosed germline cells indicates that fusions in dpix and git egg chambers are comprised of two germline cysts fused side-by-side. (I-J) Relative length (I) and width (J) of mature eggs for the indicated genotypes. In (I) and (J) sample sizes are: wt, n = 20; dpix, n = 33; git, n = 13. Statistical tests are ANOVA with post hoc Tukey’s test, and error bars are standard deviation. Significance: **** = p < 0.0001; ns = not significant. (TIF) [file pgen.1008083.s003.tif]

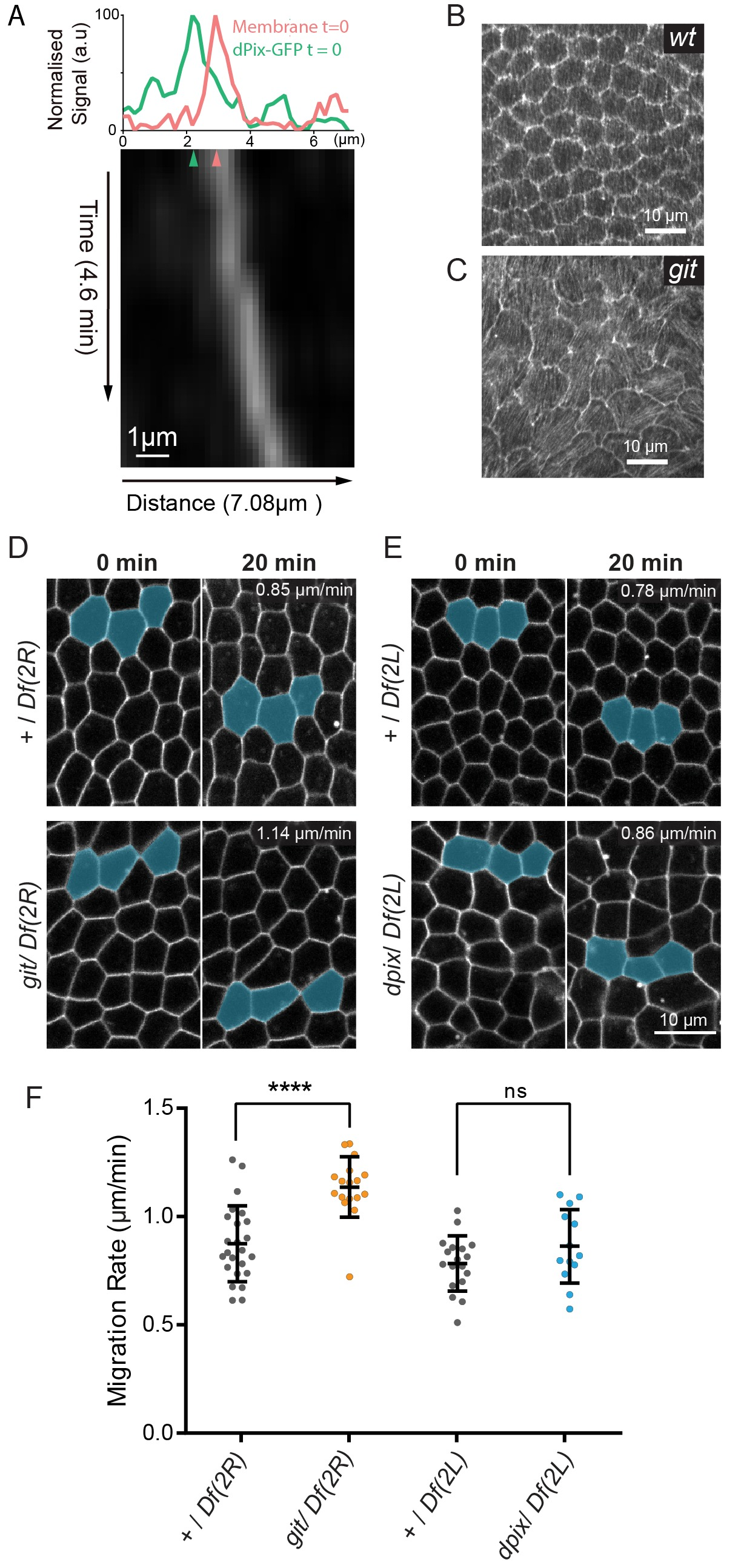

Supplement: S4 Fig — (A) Kymograph from ubi>dPix-GFP expressing egg chamber showing the direction of cell migration in relation to polarised dPix-GFP localisation. The location of polarised dPix-GFP (green arrow) relative to cell membrane (red arrow) was determined at time zero. Live imaging of the membrane of migrating cells shows the direction of movement of the leading edge membrane, and indicates that polarised dPix-GFP is positioned immediately behind the leading edge membrane. Scale bar 1 μm. (B-C) Basal actin alignment (B) is disrupted with incomplete penetrance in git (C) follicle cells. (D) Representative example of follicle cell migration (cells tracked are marked in blue) over a 20 minute period in control (+ / Df(2R)), compared to git follicle cells. (E) Representative example of follicle cell migration (cells tracked are marked in blue) over a 20 minute period in control (+ / Df(2L)), compared to dpix follicle cells. (F) Quantification of migration rates in git and dpix mutants. Statistical tests are ANOVA with post hoc Tukey’s test. Genotypes and sample sizes are: + / Df(2R), n = 24; git/ Df(2R), n = 17; + / Df(2L), n = 18; Df(2L)/ dpix, n = 13. Error bars are standard deviation. Significance: **** = p < 0.0001; ns = not significant. D. melanogaster deficiency stocks used for control and experimental genotypes in (D-F) are: Df(2R) = Df(2R)BSC595; Df(2L) = Df(2L)ED1315. (TIF) [file pgen.1008083.s004.tif]

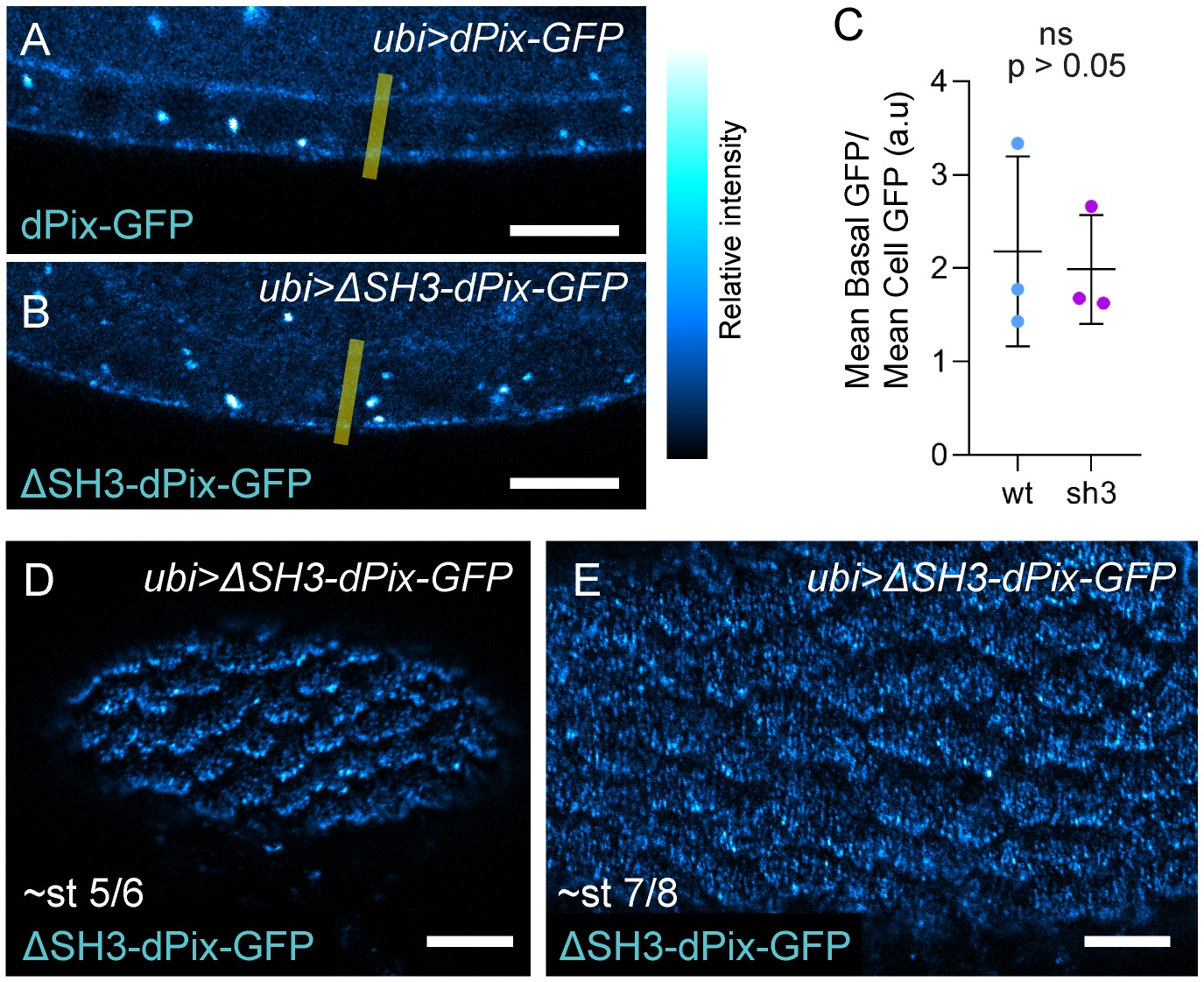

Supplement: S5 Fig — (A-B) Examples of dPix-GFP (A) and ΔSH3-dPix-GFP (B) localisation in follicular epithelium, with illustrative example of transect used for intensity quantification (yellow line). Scale bars 10 μm. Colour key indicates relative intensity of GFP signal. (C) Quantification of basal enrichment of GFP signal in dPix-GFP (wt) and ΔSH3-dPix-GFP (sh3). Statistical test is Student’s t-test, error bars are standard deviation. n = 3 egg chambers per genotype. Significance: ns = not significant. (D-E) Examples of basal planar polarised enrichment of ΔSH3-dPix-GFP protein at the indicated egg chamber stages. Scale bars are 10 μm. Genotypes are: ubi>dPix-GFP = ubi:dPix-GFP dpix p1036 (A, C); ubi>ΔSH3-dPix-GFP = ubi:ΔSH3-dPix-GFP dpix p1036 (B-E). (TIF) [file pgen.1008083.s005.tif]
